# Accounting for expected attrition in the planning of cluster randomized trials for assessing treatment effect heterogeneity

**DOI:** 10.1186/s12874-023-01887-8

**Published:** 2023-04-06

**Authors:** Jiaqi Tong, Fan Li, Michael O. Harhay, Guangyu Tong

**Affiliations:** 1 Department of Biostatistics, Yale School of Public Health, 135 College Street, CT New Haven, 06510 USA; 2 Center for Methods in Implementation and Prevention Science, Yale School of Public Health, New Haven, CT USA; 3 Department of Biostatistics, Epidemiology and Informatics, Perelman School of Medicine, University of Pennsylvania, Philadelphia, PA USA

**Keywords:** Heterogeneity of treatment effect, Missing data, Missing at random, Missing completely at random, Power calculation, Intracluster correlation coefficient, Cluster randomized trial

## Abstract

**Background:**

Detecting treatment effect heterogeneity is an important objective in cluster randomized trials and implementation research. While sample size procedures for testing the average treatment effect accounting for participant attrition assuming missing completely at random or missing at random have been previously developed, the impact of attrition on the power for detecting heterogeneous treatment effects in cluster randomized trials remains unknown.

**Methods:**

We provide a sample size formula for testing for a heterogeneous treatment effect assuming the outcome is missing completely at random. We also propose an efficient Monte Carlo sample size procedure for assessing heterogeneous treatment effect assuming covariate-dependent outcome missingness (missing at random). We compare our sample size methods with the direct inflation method that divides the estimated sample size by the mean follow-up rate. We also evaluate our methods through simulation studies and illustrate them with a real-world example.

**Results:**

Simulation results show that our proposed sample size methods under both missing completely at random and missing at random provide sufficient power for assessing heterogeneous treatment effect. The proposed sample size methods lead to more accurate sample size estimates than the direct inflation method when the missingness rate is high (e.g., ≥ 30%). Moreover, sample size estimation under both missing completely at random and missing at random is sensitive to the missingness rate, but not sensitive to the intracluster correlation coefficient among the missingness indicators.

**Conclusion:**

Our new sample size methods can assist in planning cluster randomized trials that plan to assess a heterogeneous treatment effect and participant attrition is expected to occur.

**Supplementary Information:**

The online version contains supplementary material available at 10.1186/s12874-023-01887-8.

## Contributions to the literature

- No previous studies have formally investigated how attrition can affect the sample size estimation in cluster randomized trials when the objective is to assess treatment effect heterogeneity.
- We provide a sample size formula for testing a heterogeneous treatment effect assuming the outcome is missing completely at random.
- We describe an efficient Monte Carlo sample size procedure for assessing a heterogeneous treatment effect assuming covariate-dependent outcome missingness.
- We found that the intracluster correlation coefficient among the missingness indicators has a limited impact on the power of heterogeneous treatment effect analysis in cluster randomized trials.

## Background

Cluster randomized trials (CRTs) correspond to a study design that allocate intervention at the group or community level and are increasingly popular in implementation science research [1, 2]. An essential component in planning CRTs is to estimate the sample size that provides sufficient statistical power for detecting a clinically meaningful effect size [3, 4]. When randomizing clusters, the intracluster correlation coefficient (ICC)—a quantity that measures the similarity in outcomes among units within the same cluster—is a driving factor for variance inflation and must be accounted for [5]. There has been a growing interest in studying the heterogeneous treatment effects (HTE) in clinical and public health research, particularly for studies with a health equity objective [6, 7]. Broadly, HTE refers to the differences in treatment effects across different subpopulations defined by levels of baseline covariates or effect modifiers (e.g., age, sex, education) and is modeled through treatment by covariate interaction terms. While there are different types of HTE in CRTs, our focus in this article is the systematic HTE that can be explained by measured baseline covariates, rather than the unexplained HTE across clusters that may be accounted for by a random treatment effect [8]. Accounting for treatment effect heterogeneity is important in CRTs for several considerations. First, an interaction term representing treatment effect heterogeneity in the analytical model is essential for testing and estimating differential treatment effects in patient subpopulations. Second, Tong et al. [9, 10] and Li et al. [11] have demonstrated in different CRT designs that accounting for treatment effect heterogeneity can lead to a more efficient average treatment effect estimator. In other words, failure to account for treatment effect heterogeneity could even reduce the power to study the average treatment effect.

Understanding treatment effect heterogeneity across different subgroups (e.g., age, sex, education) is not uncommon in cluster randomized trials (CRTs) [12]. For instance, according to a systematic review [13] of 64 CRTs assessing cardiovascular and chronic respiratory disease interventions, 18 out of 64 conducted the analysis with patient-level baseline covariates. As another broad example, the Consolidated Standards of Reporting Trials (CONSORT) extension in 2017 has encouraged investigators to explicitly formulate health equity objectives as the trial’s primary objective [7]. In addition, the health equity best practices guidance document [14] (item 3) developed by the National Institute on Aging (NIA) IMbedded Pragmatic Alzheimer’s disease (AD), and AD-Related Dementias (AD/ADRD) Clinical Trials (IMPACT) Collaboratory also included “*Be explicit in the sample size justifications with regard to health equity objectives*” as a recommended practice for AD/ADRD pragmatic trials (personal communication), many of which randomize nursing homes instead of individual patients. Development on HTE-based sample size procedures can therefore respond to this emerging need. Specifically, the methods developed in this work can be used in several settings. For studies whose primary interest is HTE (such as studies addressing health equity as a primary objective), our sample size methods provide tools to design a CRT with adequate power, accounting for missing outcomes. For studies whose primary interest is ATE but still hope to study HTE as a pre-specified secondary objective, our sample size tools can formally quantify the power for that secondary objective. In other words, one can assess if sufficient power can already be obtained for testing an HTE given a sample size already calculated based on the primary goal of studying ATE. For studies that are interested both in ATE and HTE, choosing a conservative sample size (maximum) from the ATE or HTE objective could be a feasible approach, in cases where there is no need for multiplicity adjustment.

Recent studies introduced methods to plan CRTs for assessing the systematic HTE with pre-specified effect modifiers such as sex and age [9, 15]. Yang et al. [15] proposed sample size methods for testing HTE in CRTs with a continuous endpoint and found that the sample size is influenced not only by the outcome ICC but also the covariate ICC, a quantity which measures the degree of similarity between effect modifiers within the same cluster [16]. Tong et al. [9] generalized their sample size procedure for unequal cluster sizes. They found that variable cluster sizes lead to negligible power loss for testing HTE with an individual-level effect modifier. The sample size procedure for assessing HTE has also been extended to accommodate three-level CRTs [11]. These developments respond to the need for CRT methods to generate knowledge on how individuals may respond differently to interventions or how the intervention may reduce existing disparity in outcomes between subgroups.

A potential limitation of existing formulas is that they assume complete follow-up of individuals and clusters, and therefore the impact of attrition on the power of the HTE test remains unknown [17–19]. Previous studies on the impact of attrition for planning CRTs focused on the average treatment effect and were typically under the missing completely at random (MCAR) assumption [20, 21]. Taljaard et al. [22] developed the sample size methods to account for expected attrition in testing the average treatment effect under MCAR, and found that the direct approach that simply inflates the sample size by mean follow-up rate can overestimate the sample size. Xu et al. [23, 24] proposed sample size methods to address outcome attrition for continuous and binary outcomes in matched-pair CRT design. Outside the CRT context, Zhu et al. [25] and Zhang et al. [26] studied attrition with the matched-pair design under the generalized estimating equations (GEE) framework and proposed a sample size formula for continuous and binary outcomes. Moreover, several studies concerning the missing data in longitudinal studies also developed sample size methods that may be applied to CRTs. For example, Roy et al. [3] developed a sample size method to address attrition in a hierarchical longitudinal design that permits differential dropout rates. Wang et al. [27] compared power methods for longitudinal data under monotone missing at random (MAR) assumption.

To date, no previous studies have formally investigated how attrition can affect the sample size estimation in CRTs when the objective is to assess treatment effect heterogeneity. This paper bridges the gap by contributing sample size procedures with outcome attrition under both the MCAR and MAR mechanisms. We provide a closed-form sample size formula for the MCAR and discuss relevant insights. For the second mechanism, we assume the effect modifier of interest is predictive for outcome attrition, and describe an efficient Monte Carlo approach for sample size estimation. The rest of the paper is organized as follows. In Methods, Testing HTE with an individual-level effect modifier section formulates the problem by introducing both the outcome model for the analysis and the missingness model. Accounting for expected attrition under MCAR section and Accounting for expected attrition under MAR section introduce our sample size methods for detecting HTE to allow for expected attrition under both MCAR and MAR. We then present simulation studies to validate our sample size procedures. Results section provides an illustration based on a real-world data example from the Work, Family, and Health Study [28]. Throughout, we compare our sample size procedures to the direct inflation approach (i.e., obtaining the sample size assuming no attrition, and then inflating it with the mean follow-up rate). Discussion section discusses the results of the simulation studies and data example. Conclusions section concludes.

## Methods

### Testing HTE with an individual-level effect modifier

We first review the typical formulation for testing confirmatory HTE in a two-arm CRT under the linear mixed model framework. We define ${Y}_{ij}$ as the continuous outcome for $i$th cluster and $j$th individual, $i\in \{1,\dots ,n\}$, $j\in \{1,\dots ,m\}$, where $n$ is the total number of clusters$; m$ is the common cluster size typically assumed in study planning. Define the cluster-level treatment indicator as ${W}_{i}$ with ${W}_{i}=1$ if a cluster is randomized to the intervention, and ${W}_{i}=0$ if randomized to the control. We focus on a single, individual-level effect modifier ${X}_{ij}$. Then, the linear mixed model accommodating the treatment by covariate interaction can be written as,

1 $$\begin{array}{c}Y_{ij}=\beta_1+\beta_2W_i+\beta_3\;X_{ij}+\beta_4W_iX_{ij}+\mu_i+\epsilon_{ij}\end{array}$$

where ${\beta }_{1}$, ${\beta }_{2}$, ${\beta }_{3}$, and ${\beta }_{4}$ are intercept, treatment main effect, covariate main effect, and treatment-by-covariate interaction effect; ${\mu }_{i}\sim N\left(0,{\sigma }_{\mu }^{2}\right)$ is the random intercept accounting for the within-cluster correlation; ${\epsilon_{ij}}\sim N\left(0, \sigma_\epsilon^2\right)$ is the residual error. The variance formula of the HTE estimator ${\widehat{\beta }}_{4}$ has been characterized in Yang et al. [15] as

$$var\left({\widehat{\beta }}_{4}\right)=\frac{{\sigma }_{y|x}^{2}\left(1-{\rho }_{y|x}\right)\{1+\left(m-1\right){\rho }_{y|x}\}}{nm{\sigma }_{w}^{2}{\sigma }_{x}^{2}\{1+\left(m-2\right){\rho }_{y|x}-\left(m-1\right){\rho }_{x}{\rho }_{y|x}\}},$$

where ${{\rho }}_{\text{x}}$ is the covariate ICC (which quantifies the ratio of between-cluster covariate variation to the total covariate variation), ${{\upsigma }}_{\text{x}}^{2}$ is the marginal variance of the effect modifier, ${{\uprho }}_{\text{y}|\text{x}}=\frac{{{\upsigma }}_{{\upmu }}^{2}}{{{\upsigma }}_{\text{y}|\text{x}}^{2}}$ is the adjusted outcome ICC (which quantifies the ratio of between-cluster outcome variation and the total outcome variance), and ${{\upsigma }}_{\text{y}|\text{x}}^{2}={{\upsigma }}_{{\upmu }}^{2}+{{\upsigma }}_{\epsilon}^{2}$ is the total adjusted variance components.

Here, ${\rho }_{x}$ is the counterpart of outcome ICC and can be defined as ${\rho }_{x}=Cov\left({X}_{ij},{X}_{ik}\right)/{\sigma }_{x}^{2}$, for $j\ne k$ where $Cov\left({X}_{ij},{X}_{ik}\right)$ represents the common covariance between effect modifiers observed for any two individuals $j$ and $k$ in a given cluster $i$. For a two-sided $z$-test with type I error rate $\alpha$ to achieve a power of $\left(1-\zeta \right)$, the required number of clusters for testing a pre-specified effect size of $\delta$ is,

2 $$n=\frac{{\left({z}_{1-\alpha /2}+{z}_{1-\zeta }\right)}^{2}{\sigma }_{y|x}^{2}\left(1-{\rho }_{y|x}\right)\left\{1+\left(m-1\right){\rho }_{y|x}\right\}}{m{\delta }^{2}{\sigma }_{w}^{2}{\sigma }_{x}^{2}\left\{1+\left(m-2\right){\rho }_{y|x}-\left(m-1\right){\rho }_{x}{\rho }_{y|x}\right\}}$$

where ${z}_{q}$ is the $q$-quantile of the standard normal distribution, and ${\sigma }_{w}^{2}$ is the Bernoulli variance of the treatment indicator. Under a balanced 1:1 randomization, ${\sigma }_{w}^{2}=1/4$.

The above sample size procedure has been extended to scenarios with randomly varying cluster sizes [9]. Assume the cluster sizes follow from a common distribution of $m_{i} \sim f(m_{i})$ with finite first and second moments as $\stackrel{-}{m}$ and ${\sigma }_{m}^{2}+ \bar{m}^{2}$. We can define the coefficient of variation (CV) of cluster sizes as, $\text{C}\text{V}={\sigma }_{m}/\stackrel{-}{m}$ [29–32]. Tong et al. [9] derived a multiplicative correction factor (CF) for the sample size requirement of HTE test with a continuous outcome as a function of the mean cluster size and CV,

3 $$\text{C}\text{F}\left(\stackrel{-}{m},CV\right)={\left[1-\text{C}{\text{V}}^{2}\frac{\stackrel{-}{m}{\rho }_{y|x}\left(1-{\rho }_{y|x}\right)\left({\rho }_{x}-{\rho }_{y|x}\right)}{\left\{1+\left(\stackrel{-}{m}-2\right){\rho }_{y|x}-\left(\stackrel{-}{m}-1\right){\rho }_{x}{\rho }_{y|x}\right\}{\left\{1+\left(\stackrel{-}{m}-1\right){\rho }_{y|x}\right\}}^{2}}\right]}^{-1}$$

The impact of this multiplicative correction factor depends on the relative size of ${\rho }_{x}$ and ${\rho }_{y|x}.$ It is equal to one if ${\rho }_{x}={\rho }_{y|x}$, below one if ${\rho }_{x}>{\rho }_{y|x}$, and above one if ${\rho }_{x}<{\rho }_{y|x}$. As shown numerically in Fig. 1 in Tong et al. [9], this correction factor is almost always 1 with a small CV of cluster size (CV = 0.3). With an extreme CV of cluster size (CV = 0.9), when the cluster size is 100, it is frequently close to 1 except when the covariate ICC approaches one. However, when the cluster size becomes smaller (e.g., 20), the correction factor is only close to 1 when the covariate ICC falls below 0.5 across a common range of outcome ICC (from 0 to 0.2). Therefore, $\text{C}\text{F}\left(\stackrel{-}{m},CV\right)$ in general has little impact on the sample size requirement.

**Fig. 1:**
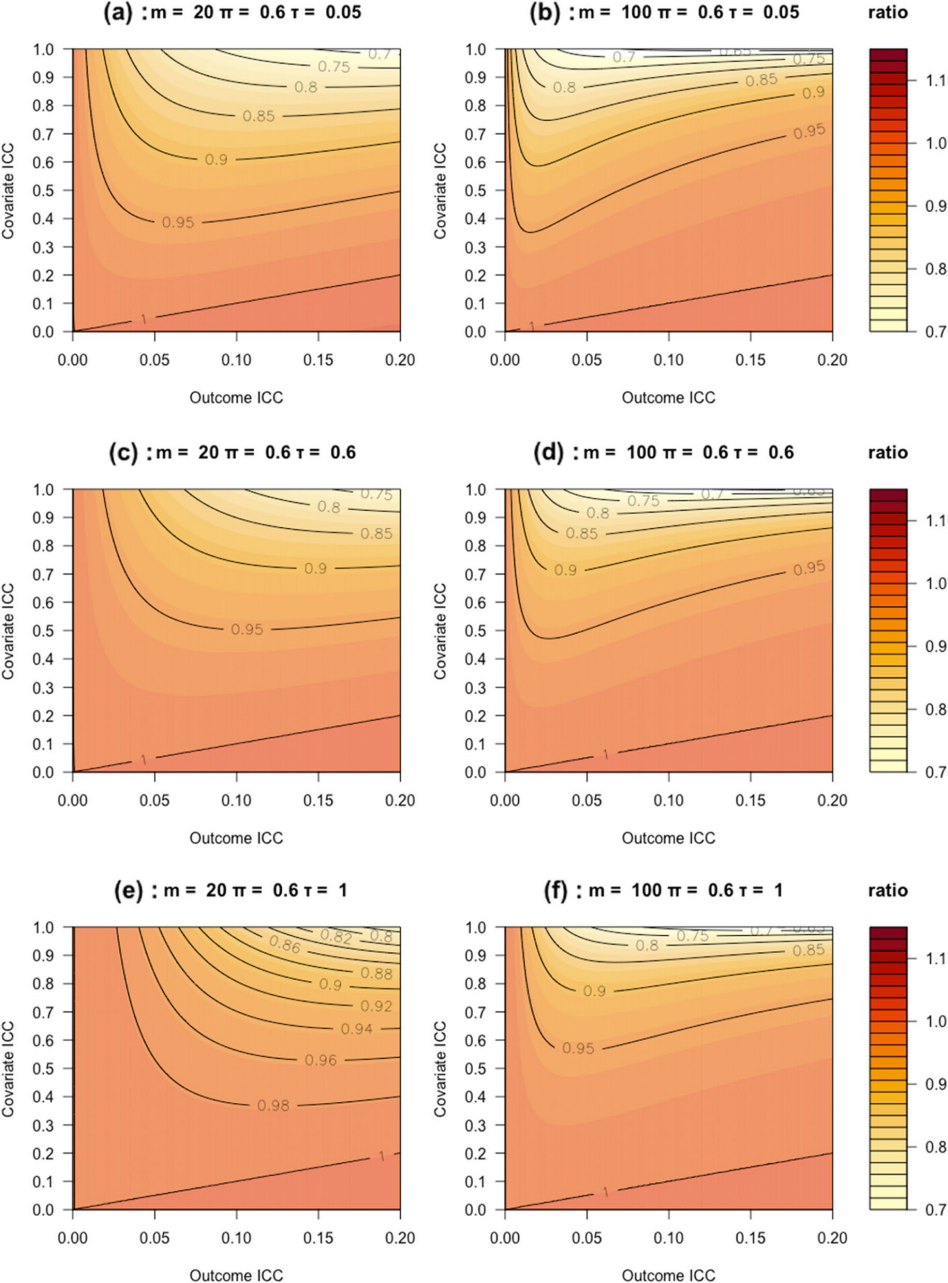
Heatmap of the ratio of sample size estimated based on the proposed formula under MCAR to that obtained from the direct inflation method under the follow-up rate of $\pi =0.6.$, the cluster size of $m\in \left\{20, 100\right\}$ and the missingness ICC of $\tau \in \left\{\text{0.05,0.6,1}\right\}$

### Accounting for expected attrition under MCAR

We propose to modify the above sample size procedure under expected attrition. Assuming a binary missingness indicator ${O}_{ij}$ such that ${O}_{ij}=1$ if the outcome ${Y}_{ij}$ is observed and ${O}_{ij}=0$ if the outcome is missing. We assume the proportion of observed outcomes or average follow-up rate as ${Pr}\left({O}_{ij}=1\right)=\pi$. Because the missingness is likely correlated within the same cluster due to cluster randomization, we assume a compound symmetric correlation structure of missingness similar to Taljaard et al. [22] The ICC between $j$th and $k$th individual missingness within $i$th cluster is defined as $corr\left({O}_{ij},{O}_{ik}\right)=\tau$ for $j\ne k$; the ICC between two individual missingness indicators in different clusters as $corr\left({O}_{ij},{O}_{{i}^{{\prime }}k}\right)=0$ for $i\ne {i}^{{\prime }}$; and by definition the ICC with itself is $corr\left({O}_{ij},{O}_{ij}\right)=1$. To ensure that the correlation matrix for the missingness indicator is positive semidefinite, $\tau$ is bounded with $-1/(m-1)\le \tau \le$1 [33]. Importantly, the lower bound is reached when the missingness is independent at the individual level, whereas the upper bound is reached when the missingness indicator for all individuals within a cluster takes the same value (cluster attrition) [22]. Therefore, this formulation accommodates the loss to follow up at both the individual and cluster levels.

Under MCAR, the missingness is independent from both outcome and covariate, such that ${O}_{ij}\perp \{{Y}_{ij},{X}_{ij},{W}_{i}$}. We have the number of observed outcomes in each cluster as ${m}_{i}=\sum _{j=1}^{m}{O}_{ij}$. The expected number of observed outcomes (observed cluster size) is ${m}^{c}=\sum _{j=1}^{m}Pr({O}_{ij}=1)=\pi m$, and the variance of the observed cluster size for each cluster is ${\sigma }_{{m}^{c}}^{2}=\sum _{j=1}^{m}Var\left({O}_{ij}\right)+{\sum }_{j\ne {j}^{{\prime }}}Cov\left({O}_{ij},{O}_{i{j}^{{\prime }}}\right)=\pi (1-\pi )m\{1+\tau (m-1\left)\right\}$. Hence the coefficient of variation of the observed cluster size becomes

$$\text{C}\text{V}={{\sigma }_{{m}^{c}}/m}^{c}=\sqrt{\frac{(1-\pi )\{1+\tau \left(m-1\right)\}}{\pi m}}$$

An important insight under MCAR is that the expected attrition leads to randomly varying cluster sizes, and therefore we can modify the formula developed in Tong et al. [9] to address attrition. Specifically, we insert $\text{C}\text{V}$ and ${m}^{c}$ into Eqs. (2) and (3), which gives,

4 $$\begin{array}{c}{n}_{1}=\frac{{\left({z}_{1-\alpha /2}+{z}_{1-\zeta }\right)}^{2}{\sigma }_{y|x}^{2}\left(1-{\rho }_{y|x}\right)\left\{1+\left(\pi m-1\right){\rho }_{y|x}\right\}}{{\pi m{\delta }^{2}\sigma }_{w}^{2}{\sigma }_{x}^{2}\left\{1+\left(\pi m-2\right){\rho }_{y|x}-\left(\pi m-1\right){\rho }_{x}{\rho }_{y|x}\right\}}\times CF\left(\pi ,\tau \right)\end{array}$$

where,

$$CF\left(\pi ,\tau \right)={\left[1-\frac{(1-\pi )\{1+\tau \left(m-1\right)\}{\rho }_{y|x}\left(1-{\rho }_{y|x}\right)\left({\rho }_{x}-{\rho }_{y|x}\right)}{\left\{1+\left(\pi m-2\right){\rho }_{y|x}-\left(\pi m-1\right){\rho }_{x}{\rho }_{y|x}\right\}{\left\{1+\left(\pi m-1\right){\rho }_{y|x}\right\}}^{2}}\right]}^{-1}$$

Here, $\text{C}\text{F}\left(\pi ,\tau \right)$ is the multiplicative correction factor of sample size under MCAR. Interestingly, the correlation between missingness only enters the formula through $\text{C}\text{F}\left(\pi ,\tau \right)$, which increases with $\tau$, suggesting the power loss is larger when the missingness correlation is higher. In two special cases, the lower bound of $\tau$ is $-1/(m-1)$, and $\text{C}\text{F}\left(\pi ,\tau \right)=1$ when this lower bound of $\tau$ is reached, which indicates the missingness modifies the cluster size by multiplying the mean follow-up rate; the upper bound of $\tau$ is $1$, where the maximum of $\text{C}\text{F}\left(\pi ,\tau \right)$ is reached (cluster attrition). However, the correction factor often takes values close to one, [9] and therefore, we anticipate that $\tau$ only minimally affects the sample size with an individual-level effect modifier given fixed $\pi$.

We compare the sample size formula in Eq. (4) to the direct approach that inflates the sample size results in Eq. (2) by the average follow-up rate $\pi$ as

$$\begin{array}{c}{n}_{0}=\frac{1}{\pi }\times \frac{{\left({z}_{1-\alpha /2}+{z}_{1-\zeta }\right)}^{2}{\sigma }_{y|x}^{2}\left(1-{\rho }_{y|x}\right)\left\{1+\left(m-1\right){\rho }_{y|x}\right\}}{m{\delta }^{2}{\sigma }_{w}^{2}{\sigma }_{x}^{2}\left\{1+\left(m-2\right){\rho }_{y|x}-\left(m-1\right){\rho }_{x}{\rho }_{y|x}\right\}}\end{array}$$

To facilitate the illustration, we provide contour plots in Fig. 1 that compares the new sample size formula we proposed versus the direct inflation approach assuming $m\in \left\{\text{20,100}\right\}$, $\pi =0.6$, and $\tau \in \left\{\text{0.05,0.6,1}\right\}$. The ratio of ${n}_{1}$ to ${n}_{0}$ is plotted with the outcome ICC in 0 to 0.2 and covariate ICC in 0 to 1. Several patterns emerge. First, the ratio of ${n}_{1}$ to ${n}_{0}$ is always smaller than 1 across all the panels in Fig. 1, suggesting that the direct inflation approach overestimates the sample size when $\tau$ is relatively small; this finding is consistent with that in Taljaard et al. [22] for testing the average treatment effect. However, when $\tau$ is large, the ratio becomes slightly larger, showing that the direct inflation approach would be less conservative. Second, the accuracy of the direct inflation approach is mainly driven by the outcome completion rate $\pi$, and relatively insensitive to the outcome ICC or covariate ICC. When $\pi$ is smaller, the direct inflation approach can be quite conservative. Additional scenarios of $m\in \{\text{20,100}$} and $\tau \in \left\{\text{0.05,0.6,1}\right\}$ with the follow-up rate $\pi =0.9$ are plotted in Appendix Fig. 1. The patterns are qualitatively similar.

### Accounting for expected attrition under MAR

We now consider the covariate-dependent attrition or MAR. With a specific effect modifier of interest, a general formulation of MAR, sometimes called the covariate-dependent missingness mechanism, assumes that ${O}_{ij}\perp {Y}_{ij} | \left\{{X}_{ij}, {W}_{i}\right\}$. For illustration, we only consider a scenario where the missingness only depends on the effect modifier ${O}_{ij}\perp \left\{{Y}_{ij},{W}_{i}\right\}|{X}_{ij}$ but an extension to allow for dependence on ${W}_{i}$ is also straightforward. To proceed, we specify the missingness model as, $Pr\left({O}_{ij}=1|{X}_{ij}\right)={\pi }_{ij}\left({X}_{ij}\right),$ and the correlation of missingness indicators between observations within the same cluster as $\tau \left({X}_{ij},{X}_{ik}\right)=corr\left({O}_{ij},{O}_{ik}|{X}_{ij},{X}_{ik}\right)$. Essentially, these quantities are counterparts of those in the previous section to allow for the dependence on the effect modifier.

Unlike MCAR, deriving the closed-form formula for $Var\left({\widehat{\beta }}_{4}\right)$ under MAR can be challenging due to the complicated correlation patterns between the missingness indicator and covariates. Under MAR, the attrition rate per cluster is no longer homogeneous and the observed cluster size could be correlated with covariate. Sample size formula under MAR will inevitably depend on the cluster size’s distributional assumption and its association with the covariate value. Therefore, we propose an efficient Monte Carlo approach to estimate the sample size through simulating the covariates and missing data patterns under pre-specified working models. Monte Carlo approach has been proven to be a popular and effective alternative when closed-form expression for variance is not available in sample size determination [34–36].

**Fig. 2:**
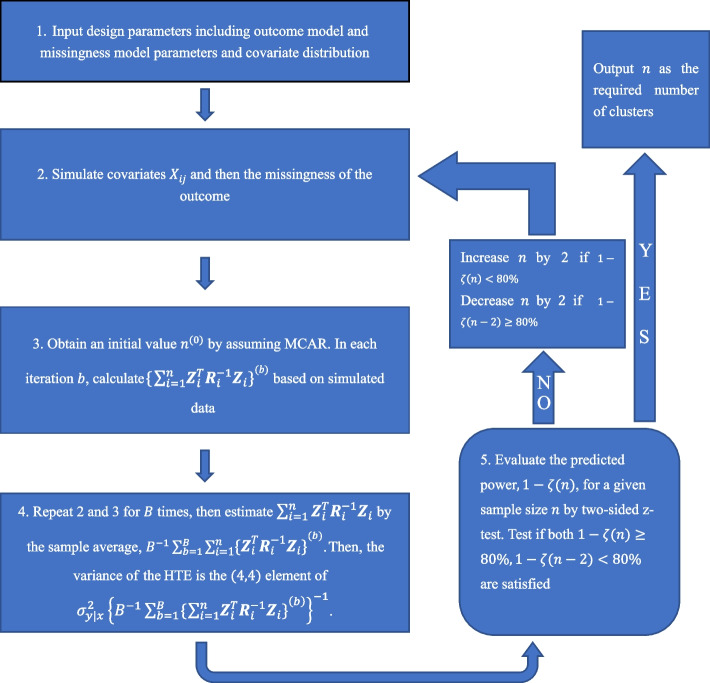
A schematic roadmap for executing the Monte Carlo approach for sample size calculation with the HTE analysis in cluster randomized trials under the missing at random assumption

The Monte Carlo sample size procedure for searching for an optimal sample size is summarized with five steps in Fig. 2. Our objective is to find the smallest number of clusters such that the predicted power is greater than or equal to a prespecified level, such as 80%. In Step 1, we specify the parameters for the outcome models, including outcome ICC, effect sizes, allocation ratio, total variances of the outcome, and the parameters for generating covariates, including covariate ICC, and other distributional parameters. For example, we can employ a mixed-effect logistic model as the missingness model with a random intercept to induce the ICC of the missingness on the logit scale [37]. Once the model configurations are determined, we set the number of simulations $B$ and an even integer ${n}^{\left(0\right)}$ as the initial number of clusters. The initial number can be obtained by assuming MCAR using Eq. (4). We iterate $B$ times of Step 2–3. In each iteration of Step 2, we simulate the effect modifier according to pre-specified distributional assumption while accounting for the covariate ICC. With a continuous effect modifier, this can be achieved via a linear mixed model; with a binary effect modifier, we can use the beta-binomial model [38] or the conditional linear family approach [39] to simulate correlated binary effect modifiers within a cluster for a pre-specified covariate ICC. In addition, we can numerically specify the intercept of the mixed-effects logistic model for missingness to set our marginal follow-up rate to the given value.

Next, we follow Yang et al. [15] and Tong et al. [9] and consider a linear mixed analysis of covariance model with a mean-centered treatment as,

$${Y}_{ij}={\beta }_{1}+{\beta }_{2} {(W}_{i}-\stackrel{-}{W})+{\beta }_{3} {X}_{ij}+{\beta }_{4}({W}_{i}-\stackrel{-}{W}){X}_{ij}+{\mu }_{i}+\epsilon_{ij}.$$

The compound symmetric correlation for cluster $i$ becomes ${\varvec{R}}_{i}=\left(1-{\rho }_{y|x}\right){\varvec{I}}_{{m}_{i}}+{\rho }_{y|x}{\varvec{J}}_{{m}_{i}}$, and its inverse is given by

5 $$\begin{array}{c}{\varvec{R}}_{i}^{-1}=\frac{1}{1-{\rho }_{y|x}}{\varvec{I}}_{{m}_{i}}-\frac{{\rho }_{y|x}}{\left(1-{\rho }_{y|x}\right)\left\{1+\left({m}_{i}-1\right){\rho }_{y|x}\right\}}{\varvec{J}}_{{m}_{i}}\end{array}$$

Here ${m}_{i}$ is the number of individuals with a measured outcome in each cluster; ${\varvec{I}}_{s}$ and ${\varvec{J}}_{s}$ are $s\times s$ identity matrix and matrix of ones, respectively. We also define the collection of design points for each individual as ${\varvec{Z}}_{ij}={\left(1,\left({W}_{i}-\stackrel{-}{W}\right),{X}_{ij},\left({W}_{i}-\stackrel{-}{W}\right){X}_{ij}\right)}^{T}$ and for each cluster as ${\varvec{Z}}_{i}={\left({\varvec{Z}}_{i1},\cdots ,{\varvec{Z}}_{i{m}_{i}}\right)}^{T}$, and $\varvec{\beta }=(\beta_1,\beta_2,\beta_3,\beta_4)^{T}$. Given values of the variance and ICC parameters, our target variance of the HTE estimator, $Var\left({\widehat{\beta }}_{4}\right)$, can be approximated by the $\left(\text{4,4}\right)$ element of ${{\sigma }_{y|x}^{2}\left\{{\sum }_{i=1}^{n}{\varvec{Z}}_{i}^{T}{\varvec{R}}_{i}^{-1}{\varvec{Z}}_{i}\right\}}^{-1}$. Since this variance is a function of $n$, we rely on the searching algorithm to solve for the required sample size. Specifically, in each iteration of Step 3, we calculate ${\sum }_{i=1}^{n}{\varvec{Z}}_{i}^{T}{\varvec{R}}_{i}^{-1}{\varvec{Z}}_{i}$ and record its realization ${\left\{{\sum }_{i=1}^{n}{\varvec{Z}}_{i}^{T}{\varvec{R}}_{i}^{-1}{\varvec{Z}}_{i}\right\}}^{\left(b\right)}$ in iteration $b,b=0, 1,\dots ,B$. In Step 4, we estimate $Var\left({\widehat{\beta }}_{4}\right)$ by the average over all iterations as ${B}^{-1}\sum _{b=1}^{B}{\left\{{\sum }_{i=1}^{n}{\varvec{Z}}_{i}^{T}{\varvec{R}}_{i}^{-1}{\varvec{Z}}_{i}\right\}}^{\left(b\right)}$ and obtain our target variance of the HTE estimator, $Var\left({\widehat{\beta }}_{4}\right)$, as the $\left(\text{4,4}\right)$ element of ${{\sigma }_{y|x}^{2}\left\{{B}^{-1}\sum _{b=1}^{B}{\left\{{\sum }_{i=1}^{n}{\varvec{Z}}_{i}^{T}{\varvec{R}}_{i}^{-1}{\varvec{Z}}_{i}\right\}}^{\left(b\right)}\right\}}^{-1}$. In Step 5, we estimate the power using a two-sided $z$-test with a pre-specified type I error rate $\alpha$ and sample size ${n}^{\left(0\right)}$. Assuming our target power is 80%, for each $k=\text{0,1},\dots$, we will assess whether the current sample size estimate ${n}^{\left(k\right)}$ ensures both $1-\zeta \left({n}^{\left(k\right)}\right)\ge 80\text{\%}$ and $1-\zeta ({n}^{\left(k\right)}-2)<80\text{\%}$, where we define the power $1-\zeta \left(n\right)$ as a function of $n$. If it does, the final sample size estimate will be ascertained as ${n}^{\left(k\right)}$ and the search concludes. Otherwise, we set ${n}^{(k+1)}={n}^{\left(k\right)}+2$ (assuming equal randomization) if the predicted power is below 80%; or decreased by 2 as ${n}^{(k+1)}={n}^{\left(k\right)}-2$ if the predicted power is over 80%.

### Simulation design under MCAR

For simplicity, our simulation only considers the balanced design with a single covariate, (i.e., $${ \sigma }_{W}^{2}=1/4$$$$, p=1$$). Since the values of $${\beta }_{1},{\beta }_{2},$$ and $${\beta }_{3}$$ do not affect the simulation results, we choose $${\beta }_{1}=0,{\beta }_{2}=0.25,$$ and $${\beta }_{3}=0.1$$. We fix the type I error rate as $$\alpha =0.0$$5, power as $$1-\zeta =80\%$$, and outcome variance $${\sigma }_{y|x}^{2}=1$$. We set the cluster size to be $$m\in \{20, 50, 100\}$$ and mean follow-up rate to be $$\pi \in \{0.7, 0.9\}$$. The values of missing indicator correlation $$\tau \in \{0.05, 0.3, 0.6, 1\}$$, in which $$1$$ indicates the special case of cluster-level attrition following Taljaard et al. [22]. We chose two outcome ICC values, $${\rho }_{y|x}\in \{0.01, 0.1\}$$; two covariate ICC values, $${\rho }_{x}\in \{0.1, 0.5\}$$. We consider both a continuous covariate and a binary covariate, where we specify the standardized effect size of $$\delta /{\sigma }_{x}=\{0.1, 0.25\}$$ for the continuous covariate, and the effect size of $$\delta =\left\{0.25, 0.45\right\}$$ for the binary covariate. The above parameter settings total $$288$$ simulation scenarios for each covariate type. The simulation code can be found at https://github.com/deckardt98/HTE_CRT_Attrition.

With a continuous covariate, we fix ${\sigma }_{x}^{2}=1$, and use the linear mixed model to simulate ${X}_{ij}=1/2+{\lambda }_{i}+{\gamma }_{ij}$ where ${\lambda }_{i}\sim N\left(0,{\rho }_{x}{\sigma }_{x}^{2}\right), { \gamma }_{ij}\sim N(0,\left(1-{\rho }_{x}\right){\sigma }_{x}^{2})$. With a binary covariate, we assume a beta-binomial distribution. The data generation follows a two-step process as follows. First, we generate the event rate ${\pi }_{i}$ for each cluster from a beta distribution, Beta $({q}_{1},{q}_{2}$). Second, we randomly generate the covariate value for each individual from $Bernoulli\left({\pi }_{i}\right)$. We fix the marginal expectation of the binary covariate as 0.3. According to the law of total expectation and law of total variance, we can solve ${q}_{1}$ and ${q}_{2}$ from ${q}_{1}/({q}_{1}+{q}_{2})=0.3$ and ${\rho }_{x}=1/(1+{q}_{1}+{q}_{2})$ simultaneously to obtain ${q}_{1}$ and ${q}_{2}$. Then the marginal variance of covariate can be obtained from ${\sigma }_{x}^{2}={q}_{1}{q}_{2}/{\left({q}_{1}+{q}_{2}\right)}^{2}$ given ${q}_{1},{q}_{2}$. Moreover, under MCAR, the missingness indicator ${O}_{ij}$ was generated using simbinCLF() function from the geeCRT package in R, which allows the specification of a common $\tau$ for all clusters to generate binary missingness indicators with a compound symmetric correlation structure in each cluster [33, 39].

We simulate each scenario as follows. (a) We first calculate the required sample size based on our proposed Eq. (4) under MCAR and round up it to the nearest even integer. (b) We generate the covariate ${X}_{ij}$ as described, and outcome ${Y}_{ij}$ from the linear mixed model in the Methods section. (c) We generate the missing indicators ${O}_{ij}$ to obtain the observed outcomes after attrition. (d) We fit the linear mixed effects model with the observed data and estimate ${\widehat{\beta }}_{4}$ via restricted maximum likelihood methods (REML) using the *nlme* package in R. (e) We calculate $p$ values under null hypothesis ${{H}_{0}:\beta }_{4}=\delta =0$ and alternative hypothesis ${{H}_{1}:\beta }_{4}=\delta \ne 0$ respectively. For each scenario, we repeat Step (a) to (e) for $3000$ times. The empirical type I error rate $\psi$ is evaluated as the proportion of false positive using the simulated data under the null, whereas the empirical power ${\varphi }_{emp}$ is evaluated as the proportion of true positive using the simulated data under the alternative. We compare empirical power ${\varphi }_{emp}$ to analytical power ${\varphi }_{pre}$ based on Equation (4) under the alternative, and empirical type I error rate $\psi$ to $0.05$ under the null. The values of ${\varphi }_{pre}$ can be slightly larger than $0.8$ because the estimated sample size is rounded to the next even integer. The corresponding Monte Carlo standard errors under $3000$ simulations assuming Bernoulli random variables are $0.004$ for the type-I error rate and $0.007$ for power. Therefore, the 95% error margin for the empirical type I error is $\pm 0.008$, and the empirical power is $\pm 0.014$. To illustrate the advantage of our methods, the sample sizes obtained from the direct inflation method are also calculated for each simulation scenario.

### Simulation design under MAR

Under MAR, we preserve the design parameters for the outcome model from the MCAR simulations but introduce additional parameters in the missingness model. We employ a mixed-effect logistic regression model to simulate the missingness indicators:

$$logit\left\{P\left({O}_{ij}=1|{X}_{ij},{b}_{i}\right)\right\}={\alpha }_{0}+{\alpha }_{1}{X}_{ij}+{b}_{i},$$

where ${b}_{i}$ is the cluster-specific intercept that follows $N(0,{\sigma }_{b}^{2})$. We fix the value of ${\alpha }_{1}$ as $0.5$ and consider one single covariate, ${X}_{ij}$, which can be either continuous or binary. The covariate data-generating procedure is identical to that under the MCAR simulation design. To make MAR simulation results comparable with MCAR, we tune the marginal follow-up rates to be $\pi \in \{0.7, 0.9\}$ by varying the value of ${\alpha }_{0}$. For the missingness ICC, $\tau$, we consider $\tau \in \{0.05, 0.3, 0.6\}$ and do not consider the extreme case of $\tau =1$. This is because $\tau$ equals to ${\sigma }_{b}^{2}/({\sigma }_{b}^{2}+{\pi }^{2}/3)$ under the mixed-effect logistic regression model [37]; in this setup, $\tau =1$ is theoretically not attainable but can be approximately once we set ${\sigma }_{b}^{2}$ to be extremely large. With the above parameter setup, we have $144$ simulation scenarios for each type of covariate. We note that both the variance $Var\left({\widehat{\beta }}_{4}\right)$ and empirical power are obtained by Monte Carlo simulations. For each scenario, we average over $B=1000$ Monta Carlo draws to obtain the variance $Var\left({\widehat{\beta }}_{4}\right)$ and calculate the sample size based on this variance. After rounding up to the nearest even integer, the analytical power is calculated from the Equation (2). We simulate $3000$ trials to calculate the empirical power following the same procedure under MCAR. To facilitate the comparison, we also calculate the required sample sizes using our proposed sample size formula under MCAR and the direct inflation method.

## Result

### Simulation results under MCAR

Table 1 shows the estimated required number of clusters for the $z$-test based on the proposed formula under MCAR given a continuous individual-level covariate. The correlation of missingness indicators is set to be $\tau =0.05$, and the missingness rate is tuned to be 0.1 or 0.3. Most scenarios have the predicted power and the empirical type I error rate within the error margin. When the number of clusters become smaller, cases with predicted power out of the error margin are occasionally observed. This is not unexpected because our sample size formula relies on the asymptotic distribution. Similar simulation results with a continuous covariate assuming $\tau \in \left\{0.3, 0.6, 1\right\}$ are included in Appendix Tables 1, 2 and 3 and another set of simulations with a binary covariate are included in Appendix Tables 4, 5, 6 and 7. Overall, our simulation verifies the accuracy of our proposed sample size formula under MCAR. In addition, compared to the sample sizes estimated via the direct inflation method, our results suggest that the magnitudes of sample size inflation, defined by the ratio of unadjusted sample size versus adjusted sample size, based on our method can be close to the follow-up rate in the direct inflation method under many simulation scenarios. However, in scenarios with large values of covariate ICC, the direct inflation method overestimates the sample size, and the degree of overestimation increases when the outcome ICC is large and when the follow-up rate is low. These findings are consistent with our numerical illustration in the Methods section. In addition, in Appendix Tables 2 and 3, we present the results for when $\tau \in \{0.3, 0.6\}$; in Appendix Table 4, we present results for the special case of $\tau =1$ where the attrition occurs at the cluster level. The results are all qualitatively similar. In general, our simulation shows that under MCAR the sample size stays approximately constant as the missingness correlation, $\tau$, varies, even when the attrition is at the cluster level ($\tau =1$). Moreover, the estimated number of clusters is insensitive to the outcome ICC when the covariate ICC is small but becomes more sensitive to the outcome ICC when the covariate ICC increases.

**Table 1:** Estimated required number of clusters for HTE test by the direct inflation (${n}_{0}$), the proposed formula under MCAR (${n}_{1}$), the empirical type I error rate of the Wald test for HTE ($\psi$), the predicted power (${\varphi }_{pre}$) and empirical power (${\varphi }_{emp}$) of the HTE test with a continuous individual-level effect modifier under MCAR. The effect size is ${\updelta }\in \{0.1, 0.25\}$. The missingness ICC is $\tau =0.05$

				$\delta =0.10$					$\delta =0.25$				
$m$	${\rho }_{x}$	${\rho }_{y|x}$	$\pi$	${n}_{0}$	${n}_{1}$	${\varphi }_{pre}$	${\varphi }_{emp}$	$\psi$	${n}_{0}$	${n}_{1}$	${\varphi }_{pre}$	${\varphi }_{emp}$	$\psi$
20	0.1	0.01	0.7	228	228	0.802	0.791	0.047	38	38	0.818	0.811	0.054
			0.9	178	178	0.803	0.815	0.052	30	30	0.823	0.797	0.052
		0.10	0.7	226	226	0.803	0.802	0.053	36	36	0.801	0.806	0.047
			0.9	176	176	0.804	0.801	0.052	28	28	0.801	0.803	0.054
	0.5	0.01	0.7	244	240	0.803	0.798	0.045	40	40	0.818	0.801	0.048
			0.9	190	190	0.804	0.796	0.054	32	32	0.824	0.781	0.051
		0.10	0.7	318	302	0.802	0.798	0.050	52	50	0.815	0.802	0.049
			0.9	248	244	0.803	0.805	0.049	40	40	0.812	0.802	0.051
50	0.1	0.01	0.7	94	92	0.801	0.793	0.049	16	16	0.832	0.829	0.048
			0.9	72	72	0.801	0.796	0.052	12	12	0.817	0.803	0.043
		0.10	0.7	90	90	0.801	0.802	0.044	16	16	0.841	0.836	0.046
			0.9	70	70	0.801	0.791	0.048	12	12	0.828	0.824	0.050
	0.5	0.01	0.7	108	104	0.804	0.799	0.045	18	18	0.834	0.787	0.065
			0.9	84	84	0.808	0.808	0.049	14	14	0.824	0.786	0.060
		0.10	0.7	144	138	0.804	0.796	0.055	24	22	0.802	0.800	0.055
			0.9	112	110	0.802	0.815	0.049	18	18	0.811	0.808	0.050
100	0.1	0.01	0.7	48	48	0.812	0.813	0.049	8	8	0.827	0.809	0.057
			0.9	38	38	0.816	0.820	0.053	6	6	0.811	0.799	0.053
		0.10	0.7	46	46	0.810	0.811	0.052	8	8	0.841	0.831	0.051
			0.9	36	36	0.812	0.814	0.048	6	6	0.828	0.829	0.057
	0.5	0.01	0.7	60	58	0.811	0.788	0.054	10	10	0.839	0.793	0.061
			0.9	48	46	0.804	0.800	0.053	8	8	0.836	0.792	0.060
		0.10	0.7	76	74	0.804	0.814	0.056	14	12	0.809	0.817	0.051
			0.9	60	60	0.812	0.818	0.047	10	10	0.828	0.823	0.054

**Table 2:** Estimated required number of clusters for HTE test by the direct inflation (${n}_{0}$), the proposed formula under MCAR (${n}_{1}$), and the proposed procedure under MAR (${n}_{2}$), the empirical type I error rate of the Wald test for HTE ($\psi$), and predicted power (${\varphi }_{pre}^{,MAR}$) and empirical power (${\varphi }_{emp}^{MAR}$) of the HTE test with a continuous individual-level effect modifier under MAR. The effect size is ${\updelta }=\{0.1, 0.25\}$. The missingness ICC is $\tau =0.05$

				$\delta =0.10$						$\delta =0.25$					
$m$	${\rho }_{x}$	${\rho }_{y|x}$	$\pi$	${n}_{0}$	${n}_{1}$	${n}_{2}$	${\varphi }_{pre}^{MAR}$	${\varphi }_{emp}^{MAR}$	$\psi$	${n}_{0}$	${n}_{1}$	${n}_{2}$	${\varphi }_{pre}^{MAR}$	${\varphi }_{emp}^{MAR}$	$\psi$
20	0.1	0.01	0.7	228	228	238	0.802	0.807	0.053	38	38	38	0.802	0.793	0.050
			0.9	178	178	182	0.803	0.804	0.051	30	30	30	0.814	0.809	0.052
		0.10	0.7	226	226	234	0.801	0.781	0.052	36	36	38	0.807	0.808	0.051
			0.9	176	176	178	0.800	0.794	0.048	28	28	30	0.820	0.816	0.051
	0.5	0.01	0.7	244	240	250	0.803	0.799	0.059	40	40	40	0.802	0.765	0.043
			0.9	190	190	194	0.805	0.791	0.050	32	32	32	0.815	0.796	0.056
		0.10	0.7	318	302	312	0.802	0.796	0.057	52	50	50	0.802	0.800	0.048
			0.9	248	244	246	0.801	0.792	0.048	40	40	40	0.807	0.792	0.053
50	0.1	0.01	0.7	94	92	96	0.800	0.789	0.048	16	16	16	0.816	0.812	0.056
			0.9	72	72	74	0.804	0.815	0.056	12	12	12	0.808	0.802	0.042
		0.10	0.7	90	90	94	0.802	0.791	0.048	16	16	16	0.826	0.831	0.051
			0.9	70	70	72	0.804	0.814	0.044	12	12	12	0.820	0.813	0.051
	0.5	0.01	0.7	108	104	108	0.804	0.807	0.045	18	18	18	0.821	0.785	0.057
			0.9	84	84	84	0.802	0.795	0.048	14	14	14	0.818	0.760	0.051
		0.10	0.7	144	138	142	0.804	0.798	0.052	24	22	24	0.826	0.815	0.058
			0.9	112	110	112	0.804	0.808	0.053	18	18	18	0.806	0.807	0.053
100	0.1	0.01	0.7	48	48	50	0.812	0.811	0.049	8	8	8	0.811	0.798	0.051
			0.9	38	38	38	0.809	0.801	0.055	6	6	6	0.803	0.800	0.051
		0.10	0.7	46	46	48	0.810	0.809	0.050	8	8	8	0.826	0.812	0.052
			0.9	36	36	36	0.805	0.812	0.047	6	6	6	0.820	0.824	0.047
	0.5	0.01	0.7	60	58	60	0.809	0.809	0.048	10	10	10	0.826	0.791	0.057
			0.9	48	46	48	0.813	0.805	0.057	8	8	8	0.828	0.784	0.062
		0.10	0.7	76	74	76	0.804	0.814	0.048	14	12	14	0.856	0.851	0.053
			0.9	60	60	60	0.808	0.816	0.047	10	10	10	0.824	0.814	0.057

**Table 3:** Estimated required number of clusters for the WFHS study with $\pi \in \left\{\text{0.935,0.87,0.61}\right\}$, $\tau \in \left\{\text{0.05,0.3,0.6}\right\}$, and $\delta \in \left\{\text{0.2,0.3}\right\}$ and the methods of direct inflation (${n}_{0}$), the formula under MCAR (${n}_{1}$), and the Monte Carlo based approach under MAR (${n}_{2}$)

		$\pi =0.935$			$\pi =0.87$			$\pi =0.61$		
$\delta$	$\tau$	${n}_{0}$	${n}_{1}$	${n}_{2}$	${n}_{0}$	${n}_{1}$	${n}_{2}$	${n}_{0}$	${n}_{1}$	${n}_{2}$
$0.2$	0.05	16	16	16	18	18	18	24	24	26
	0.3	16	16	16	18	18	18	24	24	26
	0.6	16	16	16	18	18	18	24	24	26
$0.3$	0.05	8	8	8	8	8	8	12	12	12
	0.3	8	8	8	8	8	8	12	12	12
	0.6	8	8	8	8	8	8	12	12	12

### Simulation results under MAR

Table 2 presents the estimated required number of clusters for the $z$-test based on the Monte Carlo method under MAR with a continuous covariate, $\tau =0.05$, and $\pi =0.7, 0.9$. Overall, the accuracy of our Monte Carlo-based method under MAR is confirmed as the predicted power for most of the scenario is within the error margin of the empirical power while the test maintains a valid empirical type I error rate. Only when the number of clusters becomes very small can we find empirical power lower than the predicted power. Compared to the other methods, we found that the MCAR method often underestimates the sample size when the true missingness model is MAR, especially when the pre-specified effect size is small, the cluster size is small, and the missingness rate is high. In the worst cases with lowest follow-up rate of $0.7$ and smallest cluster size of $20$, the estimated number of clusters using our formula under MCAR are $10$ clusters fewer compared to the more accurate estimation based on the proposed Monte Carlo method under MAR. As for the direct inflation method, it can underestimate the sample size when the true missingness mechanism is MAR, especially when the pre-specified effect size is small. However, when the covariate ICC and outcome ICC are large, and the missing rate is high, the direct inflation method can also overestimate the sample size. Similar results are observed for scenarios with a continuous covariate when $\tau \in \{0.3, 0.6\}$ (in Appendix Tables 8 and 9) as well as the scenarios with a binary covariate when $\tau \in \{0.05, 0.3, 0.6\}$. (Appendix Tables 10, 11 and 12). Note that with a binary covariate, the sample size formula under MCAR and the direct inflation method tend to overestimate the sample size when the true missingness mechanism is MAR.

### Application to the Work, Family, and Health Study

We demonstrate our proposed sample size methods using data from the Work, Family, and Health Study (WFHS) [28, 40]. WFHS implemented a social experiment among employees in a Fortune 500 information technology company and studied the effect of altered workplace practices and policies on work-life balance of employees. Randomization was at the group level, where each group comprised employees governed by the same leadership.

There were 799 participants nested within 56 groups enrolled in this trial at baseline and 694 participants completed the followed-up assessment at 6 months ($\pi =0.87$). The group size varies between $7$ and $60$ with an average of 29.

In this illustration, the outcome of interest is control over working hours (CWH) assessed at 6 months through survey interview. It is a continuous outcome measuring the degree of flexibility for managing working hours and was also measured at the baseline. Our goal is to estimate the sample size for studying HTE with the covariate of CWH at baseline on the CWH outcome at 6 months. According to published results [40], the outcome ICC is estimated to be 0.14; the estimated total outcome variance conditional on the baseline CWH is ${\sigma }_{y|x}^{2}\approx 0.23$; the estimated marginal variance of covariate is ${\sigma }_{x}^{2}=0.4$; the estimated ICC for the baseline CWH is ${\rho }_{x}=0.058$. The allocation ratio is 1:1 and ${\sigma }_{W}^{2}=0.25$. We consider the effect size on the outcome $\delta \in \{0.2, 0.3\}$; the correlation in the missingness $\tau \in \{0.05, 0.3, 0.6\}$. Besides $\pi =0.87$, we also consider $\pi =\{0.935, 0.610$} to expand our illustration. We estimate the required sample size under the MCAR and MAR assumptions with the same configurations of outcome and covariates ICCs and missingness ICC and marginal average missing rate. For the latter, we employ the same mixed-effects logistic model for the missingness indicator on covariate as in the Simulation design under MAR section with a slope of ${\alpha }_{1}=0.5$. We round up the calculated sample size to the nearest even integer. All calculations performed under type I error rate of 0.05 and power of 80%.

Table 3 summarizes the estimated number of clusters to test HTE with baseline CWH under different missingness mechanisms. The estimated number of clusters ranges from 16 to 26 when $\delta =0.2$, and from 8 to 12 when $\delta =0.3.$ The sample size is invariant with regard to assumptions on $\tau$ when other parameters are fixed, while being much more sensitive to the missingness rate as the number of required clusters increases when the follow-up rate is lower. For $\delta =0.3$ and under high attrition ($\pi =0.61$), the direct inflation method and the estimation formula under MCAR may underestimate the sample size when the actual missingness mechanism is MAR. When $\delta =0.3$, the estimated sample sizes are relatively invariant across different methods because the estimated sample size is generally quite small. Overall, these patterns are consistent with the findings observed in our simulation studies.

## Discussion

This paper developed new sample size procedures for assessing HTE in CRTs with outcome attrition under both MCAR and MAR mechanisms. Under MCAR, we proposed a closed-form formula for sample size calculation by adapting the result from Tong et al. [9]. This closed-form sample size is easy to implement and clarifies how design parameters can influence the sample size requirement under MCAR. Under MAR, we described a Monte Carlo method to calculate sample size. Our simulation studies show adequate performance of our proposed sample size methods under both missingness mechanisms. We also compared the performance of our sample size methods to the direct inflation method. Although the estimated sample sizes are similar across different methods in our simulation studies with a limited number of scenarios, they are expected to be more different under other settings. For example, under MCAR, Fig. 1 illustrates that the direct inflation method becomes more conservative with an increasing covariate ICC, but may be close to the proposed method when the covariate ICC approaches zero. Our simulation results also suggest the implications when the sample size is calculated using the direct inflation method. Based on the results in Tables 1 and 2, we find that the direct inflation method only overestimates the sample size under MCAR, but can either overestimate or underestimate the sample size under MAR depending on the missingness rate, cluster size, covariate, and outcome ICCs. In the context of CRT, clusters typically refer to communities, hospitals, or health systems, and it is often the case that the difference of one cluster can mean a significant change in the study budget and is important. Regardless of the values of design parameters, our formula and procedure still provide a useful approach to estimate the sample size required for a CRT accurately.

Although sample size calculations for CRTs can be carried out via simulation-based methods, it could be computationally intensive (due to repeatedly fitting multilevel models based on simulation data) and operationally cumbersome if one is interested in examining the study power across a wide range of design parameters. On the other hand, the availability of closed-form formulas and methods reduces the computational burden and effectively decreases the amount of effort in exploring many design scenarios. Perhaps more importantly, the closed-form formula clarifies key aspects and insights into the data-generating processes that determine the study power. For example, under the MCAR setting, our formula implies that the intracluster correlation coefficient of the missingness indicator generally has minimal impact on study power (except under whole cluster attrition), which further indicates that it may not be necessary to explore the change in sample size under varying intracluster correlation coefficient of the missingness indicator. Such insights can simplify the power analyses because one could then focus on exploring essential design parameters to assess the sensitivity of sample size results, instead of blindly exhausting all design parameters typically required in a simulation-based procedure. Finally, the closed-form sample size formula could offer knowledge on how key intracluster correlation parameters affect the study power, and inform investigators on selecting their values to obtain a conservative sample size when accurate knowledge is unavailable [15].

For the design parameters that are influential to the sample size estimation, our findings resemble those discussed in Tong et al. [9]. In brief, the required sample size increases as covariate ICC increases, and the required sample size decreases as outcome ICC increases. Regarding the missingness model parameters, under MCAR, the required sample size is not sensitive to the missingness ICC but increases almost proportionally as the missing rate increases. This finding facilitates the use of our method in practice because the missing rate is much easier to assume than the correlation between missing indicators at the design stage. Like MCAR, the required sample size increases as the missing rate increases under MAR. The choice of $\tau$ can be slightly more influential to sample size under MAR. However, when the cluster size is large, and the estimated sample size is small, the choice of $\tau$ still has limited impact on the sample size estimation under MAR. Reliable estimation of ICC parameters is essential for informing the design of future CRTs, and has been a topic of study in many previous works, see, for example, Maas and Hox [41], Ukoumunne et al. [42], Wu et al. [43], Preisser et al. [44] and Li et al. [45] under different clustered designs. Ridout et al. [46] also compared the performance of 20 ICC estimators for binary data. When routinely-collected data are available, one could use existing ICC estimators to obtain correlation parameters for study design purposes. Alternatively, several previous studies [43, 47–49] also published on empirical ICC estimates in completed CRTs under different settings and outcome types, and could inform the design of studies with similar features. Finally, when there is a lack of accurate knowledge of the ICC parameters nor existing data to inform such parameters, we recommend varying a range of ICC parameters (that are considered plausible depending on the context and primary outcome). For instance, in our data example, we conducted sensitivity analyses on sample sizes under various outcome or covariate ICCs. We did not further examine the sensitivity to the ICC of the missing indicator as this parameter, in most cases, is found to have minimal impact on study power. When there is no existing, routinely-collected data to inform study design parameters, it may be challenging to accurately simulate an MAR mechanism for sample size calculation at the design stage. However, when MAR (based on the effect modifier of interest) is suspected at the design stage, a reasonable approach is to conduct sensitivity analyses varying the association parameter (${{\upalpha }}_{1}$ in the logistic missingness model in the Simulation design under MAR section) between the effect modifier and the missingness while controlling for the overall missingness rate. This could generate a range of sample size estimates under different MAR mechanisms and can suggest a conservative sample size estimate over the range of missingness parameters considered. Nevertheless, in future work, it would be interesting to develop a modified sample size procedure that is robust to misspecification of the missingness model under the MAR setting.

Our Monte Carlo method for evaluating sample size under MAR is flexible insofar as the specification of the missingness model. Notably, a correct sample size estimation under MAR relies on the correct specification of the missingness model. The use of our method relies on the ignorability assumption. In this paper, we assumed a mixed-effects logistic model, and the regression parameter can be prespecified based on prior knowledge or estimated using existing data. Computation-wise, our Monte Carlo procedure does not require repeatedly fitting mixed-effects logistic regression models and therefore is more efficient in computing time than other Monte Carlo-based sample size methods developed for CRTs [50]. From our simulation results, we observe that, except for a few scenarios where the outcome ICC is relatively small, the deviation between predicted and empirical power is always within the expected Monte Carlo error margin (that is $\pm 0.015$ based on a target power of 80% under 3000 simulations). For this reason, we still recommend using our methods as a computationally efficient approach to explore sample size under MAR. However, if there is only a limited number of design scenarios and parameters to explore and computation allows, an alternative approach is to estimate the sample size based on the full simulation (by repeatedly fitting the linear mixed analysis of covariance model based on simulated data), which is expected to provide more accurate results.

Finally, there are several limitations of our proposed methods that we will address in future work. First, our methods only focus on a continuous outcome and may only provide an approximation when the outcome is binary or count. Second, our paper studies a univariate effect modifier, but multivariate effect modifiers can also arise in certain situations. For instance, a trial may wish to investigate HTE with a continuous effect modifier under both a linear term and a quadratic term. Third, our method does not address missing not at random (MNAR) scenario, in which case sensitivity analysis strategies warrants further development [51].

## Conclusion

Despite a growing interest in studying HTE in CRTs, no previous studies have formally investigated how attrition can affect the sample size estimation in a CRT when the objective is to assess treatment effect heterogeneity. We discussed sample size procedures for assessing HTE in CRTs with outcome attrition under MCAR and MAR mechanisms. Our simulation studies show satisfactory performance of our proposed sample size methods under both missingness mechanisms. The outcome ICC, covariate ICC and attrition rate are important input parameters for sample size determination at the design stage, but the ICC among the missingness indicators often has limited influence and can be considered as a nuisance parameter.

## Supplementary Information

**Additional file 1.**

## Data Availability

Data for the application can be found at, https://www.icpsr.umich.edu/web/DSDR/studies/36158.

## Abbreviations

CF: Correction factor
CRT: Cluster randomized trial
CV: Coefficient of variation
CWH: Control over working hours
HTE: Heterogeneous treatment effect
ICC: Intracluster correlation coefficient
MAR: Missing at random
MCAR: Missing completely at random
MNAR: Missing not at random
WFHS: Work, Family, and Health Study

## References

[1] Turner EL, Li F, Gallis JA (2017). Review of recent methodological developments in Group-Randomized trials: part 1-Design. Am J Public Health.

[2] Murray DM. Design and analysis of group-randomized trials: Monographs in Epidemiology and 1998.

[3] Roy A, Bhaumik DK, Aryal S (2007). Sample size determination for hierarchical longitudinal designs with differential attrition rates. Biometrics.

[4] Demidenko E (2008). Sample size and optimal design for logistic regression with binary interaction. Stat Med.

[5] Rutterford C, Copas A, Eldridge S (2015). Methods for sample size determination in cluster randomized trials. Int J Epidemiol.

[6] Cintron DW, Adler NE, Gottlieb LM (2022). Heterogeneous treatment effects in social policy studies: an assessment of contemporary articles in the health and social sciences. Ann Epidemiol.

[7] Welch VA, Norheim OF, Jull J (2017). CONSORT-Equity 2017 extension and elaboration for better reporting of health equity in randomised trials. BMJ.

[8] Hemming K, Taljaard M, Forbes A (2018). Modeling clustering and treatment effect heterogeneity in parallel and stepped-wedge cluster randomized trials. Stat Med.

[9] Tong G, Esserman D, Li F. Accounting for unequal cluster sizes in designing cluster randomized trials to detect treatment effect heterogeneity.Stat Med. 2022;41(8):1376-96. 10.1002/sim.9283 PMC10197222 34923655

[10] Tong G, Taljaard M, Li F. Sample size considerations for assessing treatment effect heterogeneity in randomized trials with heterogeneous intracluster correlations and variances, Statistics in Medicine. In press. 10.1002/sim.9811 37316956

[11] Li F, Chen X, Tian Z et al. Designing three-level cluster randomized trials to assess treatment effect heterogeneity. Biostatistics. 2022. 10.1093/biostatistics/kxac026. 10.1093/biostatistics/kxac026 PMC10583727 35861621

[12] Sun X, Briel M, Busse JW et al. Credibility of claims of subgroup effects in randomised controlled trials: systematic review. BMJ. 2012;344. 10.1136/bmj.e1553 22422832

[13] Starks MA, Sanders GD, Coeytaux RR (2019). Assessing heterogeneity of treatment effect analyses in health-related cluster randomized trials: a systematic review. PLoS ONE.

[14] Collaboratory NI. Best Practices for Integrating Health Equity into Embedded Pragmatic Clinical Trials for Dementia Care 2022:4.

[15] Yang S, Li F, Starks MA (2020). Sample size requirements for detecting treatment effect heterogeneity in cluster randomized trials. Stat Med.

[16] Raudenbush SW (1997). Statistical analysis and optimal design for cluster randomized trials. Psychol Methods.

[17] RUBIN DB (1976). Inference and missing data. Biometrika.

[18] Lefante JJ (1990). The power to detect differences in average rates of change in longitudinal studies. Stat Med.

[19] Lu K, Mehrotra DV, Liu G (2009). Sample size determination for constrained longitudinal data analysis. Stat Med.

[20] Fiero MH, Huang S, Oren E (2016). Statistical analysis and handling of missing data in cluster randomized trials: a systematic review. Trials.

[21] Zhang S, Ahn C (2012). Sample size calculation for time-averaged differences in the presence of missing data. Contemp Clin Trials.

[22] Taljaard M, Donner A, Klar N (2007). Accounting for expected attrition in the planning of community intervention trials. Stat Med.

[23] Xu X, Zhu H, Ahn C (2021). Sample size considerations for matched-pair cluster randomization design with incomplete observations of continuous outcomes. Contemp Clin Trials.

[24] Xu X, Zhu H, Hoang AQ (2021). Sample size considerations for matched-pair cluster randomization design with incomplete observations of binary outcomes. Stat Med.

[25] Zhu H, Xu X, Ahn C (2019). Sample size considerations for paired experimental design with incomplete observations of continuous outcomes. Stat Methods Med Res.

[26] Zhang S, Cao J, Ahn C (2018). Sample size calculation for before-after experiments with partially overlapping cohorts. Contemp Clin Trials.

[27] Wang C, Hall CB, Kim M (2015). A comparison of power analysis methods for evaluating effects of a predictor on slopes in longitudinal designs with missing data. Stat Methods Med Res.

[28] Work F, Network H. Work, Family, and Health Study (WFHS). Inter-university Consortium for Political and Social Research [distributor]; 2018.

[29] van Breukelen GJ, Candel MJ, Berger MP (2007). Relative efficiency of unequal versus equal cluster sizes in cluster randomized and multicentre trials. Stat Med.

[30] Candel MJ, Van Breukelen GJ (2010). Sample size adjustments for varying cluster sizes in cluster randomized trials with binary outcomes analyzed with second-order PQL mixed logistic regression. Stat Med.

[31] Li F, Tong G (2021). Sample size estimation for modified Poisson analysis of cluster randomized trials with a binary outcome. Stat Methods Med Res.

[32] Eldridge SM, Ashby D, Kerry S (2006). Sample size for cluster randomized trials: effect of coefficient of variation of cluster size and analysis method. Int J Epidemiol.

[33] Neuhaus JM (1992). Statistical methods for longitudinal and clustered designs with binary responses. Stat Methods Med Res.

[34] Liu W, Ye S, Barton BA (2020). Simulation-based power and sample size calculation for designing interrupted time series analyses of count outcomes in evaluation of health policy interventions. Contemp Clin Trials Commun.

[35] Snell KIE, Archer L, Ensor J (2021). External validation of clinical prediction models: simulation-based sample size calculations were more reliable than rules-of-thumb. J Clin Epidemiol.

[36] Shi Y, Lee JH (2018). Sample size calculations for group randomized trials with unequal group sizes through Monte Carlo simulations. Stat Methods Med Res.

[37] Eldridge SM, Ukoumunne OC, Carlin JB (2009). The Intra-Cluster correlation coefficient in cluster randomized trials: a review of definitions. Int Stat Rev.

[38] Li P, Redden DT (2015). Small sample performance of bias-corrected sandwich estimators for cluster-randomized trials with binary outcomes. Stat Med.

[39] Qaqish BF (2003). A family of Multivariate Binary Distributions for simulating correlated binary variables with specified marginal means and correlations. Biometrika.

[40] Bailey BE, Andridge R, Shoben AB (2020). Multiple imputation by predictive mean matching in cluster-randomized trials. BMC Med Res Methodol.

[41] Maas CJ, Hox JJ (2005). Sufficient sample sizes for multilevel modeling. Methodology.

[42] Ukoumunne OC (2002). A comparison of confidence interval methods for the intraclass correlation coefficient in cluster randomized trials. Stat Med.

[43] Wu S, Crespi CM, Wong WK (2012). Comparison of methods for estimating the intraclass correlation coefficient for binary responses in cancer prevention cluster randomized trials. Contemp Clin Trials.

[44] Preisser JS, Lu B, Qaqish BF (2008). Finite sample adjustments in estimating equations and covariance estimators for intracluster correlations. Stat Med.

[45] Li F, Yu H, Rathouz PJ (2022). Marginal modeling of cluster-period means and intraclass correlations in stepped wedge designs with binary outcomes. Biostatistics.

[46] Ridout MS, Clarice GBD, Firth D (1999). Estimating Intraclass correlation for Binary Data. Biometrics.

[47] Murray DM, Blitstein JL (2003). Methods to reduce the impact of Intraclass correlation in Group-Randomized trials. Eval Rev.

[48] Campbell MK, Fayers PM, Grimshaw JM (2005). Determinants of the intracluster correlation coefficient in cluster randomized trials: the case of implementation research. Clin Trials.

[49] Korevaar E, Kasza J, Taljaard M (2021). Intra-cluster correlations from the CLustered OUtcome dataset bank to inform the design of longitudinal cluster trials. Clin Trials.

[50] Landau S, Stahl D (2013). Sample size and power calculations for medical studies by simulation when closed form expressions are not available. Stat Methods Med Res.

[51] Tong G, Li F, Allen AS. Missing Data. In Piantadosi S, Meinert CL, editors. Principles and Practice of Clinical Trials. Cham: Springer International Publishing; 2019. p. 1–21.

