# Correction: Accounting for expected attrition in the planning of cluster randomized trials for assessing treatment effect heterogeneity

**DOI:** 10.1186/s12874-023-01932-6

**Published:** 2023-06-07

**Authors:** Jiaqi Tong, Fan Li, Michael O. Harhay, Guangyu Tong

**Affiliations:** 1 Department of Biostatistics, Yale School of Public Health, 135 College Street, New Haven, CT 06510 USA; 2 Center for Methods in Implementation and Prevention Science, Yale School of Public Health, New Haven, CT USA; 3 Department of Biostatistics, Epidemiology and Informatics, Perelman School of Medicine, University of Pennsylvania, Philadelphia, PA USA

**Correction: BMC Medical Research Methodology 23, 85 (2023)**

10.1186/s12874-023-01887-8

Following the publication of the original article [1], the authors request further changes due to editorial irregularities.

The original article [1] has been updated.
