# Supplementary material for: Accounting for expected attrition in the planning of cluster randomized trials for assessing treatment effect heterogeneity
Source: BMC Med Res Methodol. 2023 Apr 6;23:85. doi: 10.1186/s12874-023-01887-8 (PMC10077680; doi:10.1186/s12874-023-01887-8)
Supplement: Supplementary file 1 — Additional file 1. [file 12874_2023_1887_MOESM1_ESM.docx]

**APPENDIX**

**Appendix Figure 1**. Heatmap of the ratio of sample size estimated based on the proposed formula under MCAR to that obtained from the direct inflation method under the completion rate of $\pi=0.9.$, the cluster size of $m=\left\{ 20, 100 \right\}$ and the correlation in the missingness of $\tau=\left\{ 0.05,0.6,1 \right\}.$


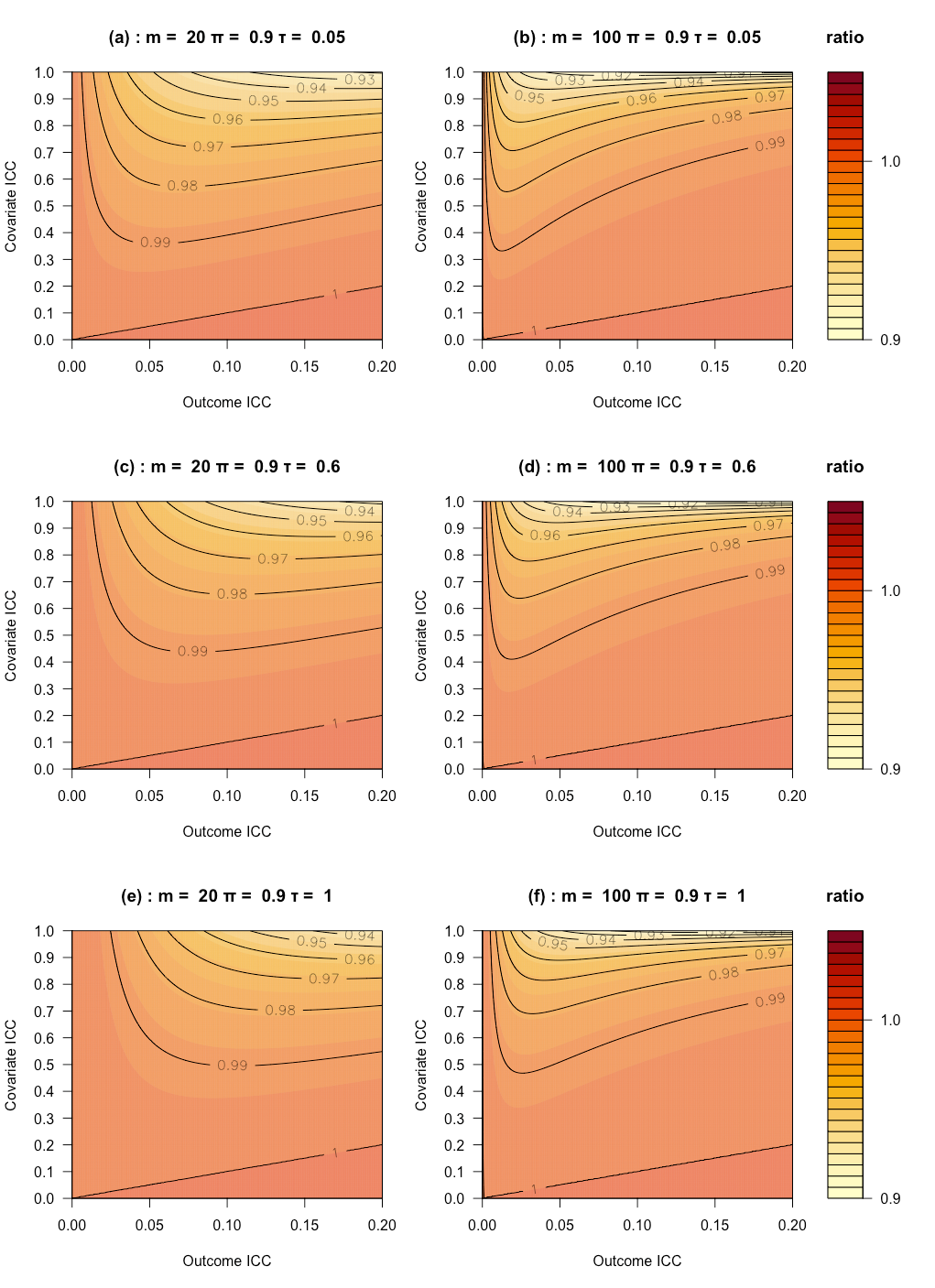


**Appendix Table 1**. Estimated required number of clusters for HTE test by the direct inflation ($n_{0}$), the proposed formula under MCAR ($n_{1}$), the empirical type I error rate of the Wald test for HTE ($\psi$), the predicted power ($\phi_{pre}$) and empirical power ($\phi_{emp}$) of the HTE test with a continuous individual-level effect modifier under MCAR. The effect size for power is $\delta\in\{0.1, 0.25\}$. The missingness ICC is $\tau=0.3$.

|  | | | | $\delta=0.10$ | | | | | $\delta=0.25$ | | | | |
| --- | --- | --- | --- | --- | --- | --- | --- | --- | --- | --- | --- | --- | --- |
| $m$ | $\rho_{x}$ | $\rho_{y\vert x}$ | $\pi$ | $n_{0}$ | $n_{1}$ | $\phi_{pre}$ | $\phi_{emp}$ | $\psi$ | $n_{0}$ | $n_{1}$ | $\phi_{pre}$ | $\phi_{emp}$ | $\psi$ |
| 20 | 0.1 | 0.01 | 0.7 | 228 | 228 | 0.802 | 0.806 | 0.046 | 38 | 38 | 0.818 | 0.816 | 0.051 |
|  |  |  | 0.9 | 178 | 178 | 0.803 | 0.805 | 0.051 | 30 | 30 | 0.823 | 0.825 | 0.049 |
|  |  | 0.10 | 0.7 | 226 | 226 | 0.803 | 0.816 | 0.049 | 36 | 36 | 0.801 | 0.802 | 0.043 |
|  |  |  | 0.9 | 176 | 176 | 0.804 | 0.795 | 0.052 | 28 | 28 | 0.801 | 0.788 | 0.044 |
|  | 0.5 | 0.01 | 0.7 | 244 | 240 | 0.801 | 0.797 | 0.048 | 40 | 40 | 0.817 | 0.796 | 0.052 |
|  |  |  | 0.9 | 190 | 190 | 0.803 | 0.791 | 0.056 | 32 | 32 | 0.823 | 0.791 | 0.049 |
|  |  | 0.10 | 0.7 | 318 | 304 | 0.802 | 0.797 | 0.048 | 52 | 50 | 0.813 | 0.798 | 0.054 |
|  |  |  | 0.9 | 248 | 244 | 0.802 | 0.804 | 0.059 | 40 | 40 | 0.812 | 0.810 | 0.046 |
| 50 | 0.1 | 0.01 | 0.7 | 94 | 92 | 0.800 | 0.798 | 0.045 | 16 | 16 | 0.832 | 0.810 | 0.047 |
|  |  |  | 0.9 | 72 | 72 | 0.801 | 0.795 | 0.051 | 12 | 12 | 0.817 | 0.806 | 0.052 |
|  |  | 0.10 | 0.7 | 90 | 90 | 0.801 | 0.802 | 0.051 | 16 | 16 | 0.841 | 0.830 | 0.057 |
|  |  |  | 0.9 | 70 | 70 | 0.801 | 0.803 | 0.055 | 12 | 12 | 0.828 | 0.813 | 0.048 |
|  | 0.5 | 0.01 | 0.7 | 108 | 104 | 0.801 | 0.779 | 0.054 | 18 | 18 | 0.831 | 0.792 | 0.058 |
|  |  |  | 0.9 | 84 | 84 | 0.807 | 0.793 | 0.051 | 14 | 14 | 0.823 | 0.777 | 0.056 |
|  |  | 0.10 | 0.7 | 144 | 138 | 0.803 | 0.807 | 0.057 | 24 | 22 | 0.801 | 0.793 | 0.052 |
|  |  |  | 0.9 | 112 | 110 | 0.802 | 0.813 | 0.049 | 18 | 18 | 0.811 | 0.800 | 0.048 |
| 100 | 0.1 | 0.01 | 0.7 | 48 | 48 | 0.811 | 0.802 | 0.053 | 8 | 8 | 0.826 | 0.803 | 0.053 |
|  |  |  | 0.9 | 38 | 38 | 0.816 | 0.823 | 0.045 | 6 | 6 | 0.811 | 0.792 | 0.050 |
|  |  | 0.10 | 0.7 | 46 | 46 | 0.810 | 0.799 | 0.047 | 8 | 8 | 0.841 | 0.815 | 0.051 |
|  |  |  | 0.9 | 36 | 36 | 0.812 | 0.800 | 0.046 | 6 | 6 | 0.828 | 0.830 | 0.042 |
|  | 0.5 | 0.01 | 0.7 | 60 | 58 | 0.808 | 0.802 | 0.053 | 10 | 10 | 0.836 | 0.790 | 0.056 |
|  |  |  | 0.9 | 48 | 46 | 0.803 | 0.793 | 0.052 | 8 | 8 | 0.835 | 0.781 | 0.064 |
|  |  | 0.10 | 0.7 | 76 | 74 | 0.803 | 0.805 | 0.046 | 14 | 12 | 0.808 | 0.792 | 0.060 |
|  |  |  | 0.9 | 60 | 60 | 0.812 | 0.817 | 0.047 | 10 | 10 | 0.828 | 0.824 | 0.053 |

**Appendix Table 2**. Estimated required number of clusters for HTE test by the direct inflation ($n_{0}$), the proposed formula under MCAR ($n_{1}$), the empirical type I error rate of the Wald test for HTE ($\psi$), the predicted power ($\phi_{pre}$) and empirical power ($\phi_{emp}$) of the HTE test with a continuous individual-level effect modifier under MCAR. The effect size for power is $\delta\in\{0.1, 0.25\}$. The missingness ICC is $\tau=0.6$.

|  | | | | $\delta=0.10$ | | | | | $\delta=0.25$ | | | | |
| --- | --- | --- | --- | --- | --- | --- | --- | --- | --- | --- | --- | --- | --- |
| $m$ | $\rho_{x}$ | $\rho_{y\vert x}$ | $\pi$ | $n_{0}$ | $n_{1}$ | $\phi_{pre}$ | $\phi_{emp}$ | $\psi$ | $n_{0}$ | $n_{1}$ | $\phi_{pre}$ | $\phi_{emp}$ | $\psi$ |
| 20 | 0.1 | 0.01 | 0.7 | 228 | 228 | 0.801 | 0.811 | 0.049 | 38 | 38 | 0.817 | 0.808 | 0.048 |
|  |  |  | 0.9 | 178 | 178 | 0.802 | 0.802 | 0.045 | 30 | 30 | 0.822 | 0.803 | 0.048 |
|  |  | 0.10 | 0.7 | 226 | 226 | 0.803 | 0.804 | 0.044 | 36 | 36 | 0.801 | 0.786 | 0.054 |
|  |  |  | 0.9 | 176 | 176 | 0.804 | 0.792 | 0.045 | 28 | 28 | 0.801 | 0.796 | 0.049 |
|  | 0.5 | 0.01 | 0.7 | 244 | 242 | 0.802 | 0.790 | 0.053 | 40 | 40 | 0.814 | 0.771 | 0.051 |
|  |  |  | 0.9 | 190 | 190 | 0.803 | 0.798 | 0.050 | 32 | 32 | 0.822 | 0.786 | 0.052 |
|  |  | 0.10 | 0.7 | 318 | 306 | 0.802 | 0.789 | 0.049 | 52 | 50 | 0.810 | 0.784 | 0.060 |
|  |  |  | 0.9 | 248 | 244 | 0.802 | 0.803 | 0.054 | 40 | 40 | 0.811 | 0.809 | 0.048 |
| 50 | 0.1 | 0.01 | 0.7 | 94 | 94 | 0.808 | 0.807 | 0.043 | 16 | 16 | 0.831 | 0.809 | 0.045 |
|  |  |  | 0.9 | 72 | 72 | 0.801 | 0.794 | 0.047 | 12 | 12 | 0.817 | 0.814 | 0.055 |
|  |  | 0.10 | 0.7 | 90 | 90 | 0.801 | 0.796 | 0.057 | 16 | 16 | 0.841 | 0.811 | 0.052 |
|  |  |  | 0.9 | 70 | 70 | 0.801 | 0.797 | 0.048 | 12 | 12 | 0.828 | 0.811 | 0.049 |
|  | 0.5 | 0.01 | 0.7 | 108 | 106 | 0.804 | 0.805 | 0.053 | 18 | 18 | 0.827 | 0.778 | 0.052 |
|  |  |  | 0.9 | 84 | 84 | 0.806 | 0.794 | 0.050 | 14 | 14 | 0.822 | 0.776 | 0.055 |
|  |  | 0.10 | 0.7 | 144 | 138 | 0.801 | 0.785 | 0.051 | 24 | 24 | 0.833 | 0.829 | 0.051 |
|  |  |  | 0.9 | 112 | 110 | 0.802 | 0.800 | 0.048 | 18 | 18 | 0.810 | 0.807 | 0.054 |
| 100 | 0.1 | 0.01 | 0.7 | 48 | 48 | 0.810 | 0.804 | 0.045 | 8 | 8 | 0.826 | 0.778 | 0.053 |
|  |  |  | 0.9 | 38 | 38 | 0.816 | 0.828 | 0.054 | 6 | 6 | 0.811 | 0.783 | 0.053 |
|  |  | 0.10 | 0.7 | 46 | 46 | 0.810 | 0.792 | 0.054 | 8 | 8 | 0.841 | 0.811 | 0.055 |
|  |  |  | 0.9 | 36 | 36 | 0.812 | 0.826 | 0.047 | 6 | 6 | 0.828 | 0.805 | 0.047 |
|  | 0.5 | 0.01 | 0.7 | 60 | 58 | 0.803 | 0.785 | 0.052 | 10 | 10 | 0.831 | 0.758 | 0.061 |
|  |  |  | 0.9 | 48 | 46 | 0.802 | 0.768 | 0.065 | 8 | 8 | 0.834 | 0.777 | 0.057 |
|  |  | 0.10 | 0.7 | 76 | 74 | 0.803 | 0.801 | 0.052 | 14 | 12 | 0.808 | 0.784 | 0.048 |
|  |  |  | 0.9 | 60 | 60 | 0.812 | 0.810 | 0.048 | 10 | 10 | 0.828 | 0.819 | 0.049 |

**Appendix Table 3**. Estimated required number of clusters for HTE test by the direct inflation ($n_{0}$), the proposed formula under MCAR ($n_{1}$), the empirical type I error rate of the Wald test for HTE ($\psi$), the predicted power ($\phi_{pre}$) and empirical power ($\phi_{emp}$) of the HTE test with a continuous individual-level effect modifier under MCAR. The effect size for power is $\delta\in\{0.1, 0.25\}$. The missingness ICC is $\tau=1$.

|  | | | | $\delta=0.10$ | | | | | $\delta=0.25$ | | | | |
| --- | --- | --- | --- | --- | --- | --- | --- | --- | --- | --- | --- | --- | --- |
| $m$ | $\rho_{x}$ | $\rho_{y\vert x}$ | $\pi$ | $n_{0}$ | $n_{1}$ | $\phi_{pre}$ | $\phi_{emp}$ | $\psi$ | $n_{0}$ | $n_{1}$ | $\phi_{pre}$ | $\phi_{emp}$ | $\psi$ |
| 20 | 0.1 | 0.01 | 0.7 | 228 | 228 | 0.801 | 0.794 | 0.055 | 38 | 38 | 0.817 | 0.791 | 0.053 |
|  |  |  | 0.9 | 178 | 178 | 0.802 | 0.794 | 0.046 | 30 | 30 | 0.822 | 0.810 | 0.060 |
|  |  | 0.10 | 0.7 | 226 | 226 | 0.803 | 0.793 | 0.055 | 36 | 36 | 0.801 | 0.782 | 0.048 |
|  |  |  | 0.9 | 176 | 176 | 0.804 | 0.810 | 0.054 | 28 | 28 | 0.801 | 0.779 | 0.052 |
|  | 0.5 | 0.01 | 0.7 | 244 | 244 | 0.802 | 0.794 | 0.056 | 40 | 40 | 0.811 | 0.770 | 0.054 |
|  |  |  | 0.9 | 190 | 190 | 0.802 | 0.796 | 0.048 | 32 | 32 | 0.821 | 0.800 | 0.052 |
|  |  | 0.10 | 0.7 | 318 | 308 | 0.801 | 0.792 | 0.054 | 52 | 50 | 0.806 | 0.784 | 0.055 |
|  |  |  | 0.9 | 248 | 244 | 0.801 | 0.803 | 0.056 | 40 | 40 | 0.810 | 0.797 | 0.058 |
| 50 | 0.1 | 0.01 | 0.7 | 94 | 94 | 0.807 | 0.794 | 0.051 | 16 | 16 | 0.830 | 0.799 | 0.057 |
|  |  |  | 0.9 | 72 | 72 | 0.801 | 0.809 | 0.053 | 12 | 12 | 0.816 | 0.817 | 0.048 |
|  |  | 0.10 | 0.7 | 90 | 90 | 0.801 | 0.808 | 0.049 | 16 | 16 | 0.841 | 0.808 | 0.054 |
|  |  |  | 0.9 | 70 | 70 | 0.801 | 0.800 | 0.060 | 12 | 12 | 0.828 | 0.804 | 0.052 |
|  | 0.5 | 0.01 | 0.7 | 108 | 108 | 0.806 | 0.799 | 0.047 | 18 | 18 | 0.822 | 0.742 | 0.061 |
|  |  |  | 0.9 | 84 | 84 | 0.805 | 0.800 | 0.052 | 14 | 14 | 0.820 | 0.770 | 0.057 |
|  |  | 0.10 | 0.7 | 144 | 140 | 0.805 | 0.781 | 0.058 | 24 | 24 | 0.831 | 0.791 | 0.050 |
|  |  |  | 0.9 | 112 | 110 | 0.801 | 0.796 | 0.053 | 18 | 18 | 0.810 | 0.803 | 0.051 |
| 100 | 0.1 | 0.01 | 0.7 | 48 | 48 | 0.809 | 0.804 | 0.043 | 8 | 8 | 0.825 | 0.770 | 0.051 |
|  |  |  | 0.9 | 38 | 38 | 0.816 | 0.805 | 0.055 | 6 | 6 | 0.811 | 0.790 | 0.052 |
|  |  | 0.10 | 0.7 | 46 | 46 | 0.810 | 0.800 | 0.048 | 8 | 8 | 0.841 | 0.792 | 0.047 |
|  |  |  | 0.9 | 36 | 36 | 0.812 | 0.818 | 0.053 | 6 | 6 | 0.828 | 0.806 | 0.052 |
|  | 0.5 | 0.01 | 0.7 | 60 | 60 | 0.810 | 0.783 | 0.053 | 10 | 10 | 0.826 | 0.726 | 0.057 |
|  |  |  | 0.9 | 48 | 46 | 0.801 | 0.777 | 0.050 | 8 | 8 | 0.832 | 0.770 | 0.063 |
|  |  | 0.10 | 0.7 | 76 | 74 | 0.802 | 0.783 | 0.053 | 14 | 12 | 0.807 | 0.774 | 0.059 |
|  |  |  | 0.9 | 60 | 60 | 0.812 | 0.799 | 0.054 | 10 | 10 | 0.828 | 0.817 | 0.046 |

**Appendix Table 4.** Estimated required number of clusters for HTE test by the direct inflation ($n_{0}$), the proposed formula under MCAR ($n_{1}$), the empirical type I error rate of the Wald test for HTE ($\psi$), the predicted power ($\phi_{pre}$) and empirical power ($\phi_{emp}$) of the HTE test with a binary individual-level effect modifier under MCAR. The effect size for power is $\delta\in\{0.25, 0.45\}$. The missingness ICC is $\tau=0.05$.

|  | | | | $\delta=0.25$ | | | | | $\delta=0.45$ | | | | |
| --- | --- | --- | --- | --- | --- | --- | --- | --- | --- | --- | --- | --- | --- |
| $m$ | $\rho_{x}$ | $\rho_{y\vert x}$ | $\pi$ | $n_{0}$ | $n_{1}$ | $\phi_{pre}$ | $\phi_{emp}$ | $\psi$ | $n_{0}$ | $n_{1}$ | $\phi_{pre}$ | $\phi_{emp}$ | $\psi$ |
| 20 | 0.1 | 0.01 | 0.7 | 174 | 174 | 0.803 | 0.802 | 0.048 | 54 | 54 | 0.805 | 0.799 | 0.047 |
|  |  |  | 0.9 | 136 | 136 | 0.804 | 0.812 | 0.050 | 42 | 42 | 0.804 | 0.789 | 0.052 |
|  |  | 0.10 | 0.7 | 172 | 172 | 0.803 | 0.797 | 0.049 | 54 | 54 | 0.809 | 0.802 | 0.046 |
|  |  |  | 0.9 | 134 | 134 | 0.803 | 0.811 | 0.047 | 42 | 42 | 0.809 | 0.808 | 0.058 |
|  | 0.5 | 0.01 | 0.7 | 186 | 182 | 0.801 | 0.790 | 0.048 | 58 | 58 | 0.813 | 0.793 | 0.047 |
|  |  |  | 0.9 | 146 | 144 | 0.802 | 0.786 | 0.052 | 46 | 46 | 0.815 | 0.795 | 0.048 |
|  |  | 0.10 | 0.7 | 242 | 230 | 0.802 | 0.789 | 0.056 | 76 | 72 | 0.808 | 0.784 | 0.053 |
|  |  |  | 0.9 | 188 | 186 | 0.803 | 0.793 | 0.048 | 58 | 58 | 0.807 | 0.805 | 0.049 |
| 50 | 0.1 | 0.01 | 0.7 | 72 | 70 | 0.800 | 0.795 | 0.047 | 22 | 22 | 0.807 | 0.807 | 0.045 |
|  |  |  | 0.9 | 56 | 56 | 0.809 | 0.808 | 0.050 | 18 | 18 | 0.825 | 0.809 | 0.047 |
|  |  | 0.10 | 0.7 | 70 | 70 | 0.809 | 0.817 | 0.057 | 22 | 22 | 0.816 | 0.819 | 0.059 |
|  |  |  | 0.9 | 54 | 54 | 0.806 | 0.801 | 0.053 | 18 | 18 | 0.835 | 0.824 | 0.048 |
|  | 0.5 | 0.01 | 0.7 | 82 | 80 | 0.808 | 0.787 | 0.052 | 26 | 26 | 0.828 | 0.813 | 0.051 |
|  |  |  | 0.9 | 64 | 64 | 0.808 | 0.802 | 0.050 | 20 | 20 | 0.813 | 0.777 | 0.053 |
|  |  | 0.10 | 0.7 | 110 | 106 | 0.807 | 0.794 | 0.060 | 34 | 34 | 0.821 | 0.800 | 0.054 |
|  |  |  | 0.9 | 86 | 84 | 0.803 | 0.799 | 0.037 | 28 | 26 | 0.804 | 0.783 | 0.047 |
| 100 | 0.1 | 0.01 | 0.7 | 36 | 36 | 0.805 | 0.801 | 0.048 | 12 | 12 | 0.834 | 0.822 | 0.062 |
|  |  |  | 0.9 | 28 | 28 | 0.803 | 0.806 | 0.056 | 10 | 10 | 0.857 | 0.845 | 0.054 |
|  |  | 0.10 | 0.7 | 36 | 36 | 0.820 | 0.816 | 0.047 | 12 | 12 | 0.848 | 0.847 | 0.045 |
|  |  |  | 0.9 | 28 | 28 | 0.820 | 0.819 | 0.050 | 10 | 10 | 0.871 | 0.873 | 0.046 |
|  | 0.5 | 0.01 | 0.7 | 46 | 44 | 0.810 | 0.812 | 0.045 | 14 | 14 | 0.821 | 0.775 | 0.060 |
|  |  |  | 0.9 | 36 | 36 | 0.815 | 0.799 | 0.050 | 12 | 12 | 0.843 | 0.785 | 0.062 |
|  |  | 0.10 | 0.7 | 58 | 56 | 0.801 | 0.800 | 0.050 | 18 | 18 | 0.817 | 0.784 | 0.052 |
|  |  |  | 0.9 | 46 | 46 | 0.815 | 0.798 | 0.051 | 14 | 14 | 0.809 | 0.776 | 0.047 |

**Appendix Table 5.** Estimated required number of clusters for HTE test by the direct inflation ($n_{0}$), the proposed formula under MCAR ($n_{1}$), the empirical type I error rate of the Wald test for HTE ($\psi$), the predicted power ($\phi_{pre}$) and empirical power ($\phi_{emp}$) of the HTE test with a binary individual-level effect modifier under MCAR. The effect size for power is $\delta\in\{0.25, 0.45\}$. The missingness ICC is $\tau=0.3$.

|  | | | | $\delta=0.25$ | | | | | $\delta=0.45$ | | | | |
| --- | --- | --- | --- | --- | --- | --- | --- | --- | --- | --- | --- | --- | --- |
| $m$ | $\rho_{x}$ | $\rho_{y\vert x}$ | $\pi$ | $n_{0}$ | $n_{1}$ | $\phi_{pre}$ | $\phi_{emp}$ | $\psi_{0}$ | $n_{0}$ | $n_{1}$ | $\phi_{pre}$ | $\phi_{emp}$ | $\psi_{0}$ |
| 20 | 0.1 | 0.01 | 0.7 | 174 | 174 | 0.803 | 0.803 | 0.050 | 54 | 54 | 0.805 | 0.792 | 0.052 |
|  |  |  | 0.9 | 136 | 136 | 0.804 | 0.815 | 0.048 | 42 | 42 | 0.804 | 0.790 | 0.049 |
|  |  | 0.10 | 0.7 | 172 | 172 | 0.803 | 0.792 | 0.051 | 54 | 54 | 0.809 | 0.804 | 0.060 |
|  |  |  | 0.9 | 134 | 134 | 0.803 | 0.802 | 0.052 | 42 | 42 | 0.809 | 0.809 | 0.057 |
|  | 0.5 | 0.01 | 0.7 | 186 | 184 | 0.803 | 0.788 | 0.053 | 58 | 58 | 0.811 | 0.807 | 0.056 |
|  |  |  | 0.9 | 146 | 144 | 0.801 | 0.794 | 0.048 | 46 | 46 | 0.815 | 0.796 | 0.054 |
|  |  | 0.10 | 0.7 | 242 | 232 | 0.803 | 0.783 | 0.058 | 76 | 72 | 0.805 | 0.786 | 0.054 |
|  |  |  | 0.9 | 188 | 186 | 0.802 | 0.795 | 0.052 | 58 | 58 | 0.806 | 0.796 | 0.056 |
| 50 | 0.1 | 0.01 | 0.7 | 72 | 72 | 0.810 | 0.811 | 0.053 | 22 | 22 | 0.807 | 0.800 | 0.056 |
|  |  |  | 0.9 | 56 | 56 | 0.809 | 0.802 | 0.050 | 18 | 18 | 0.825 | 0.805 | 0.059 |
|  |  | 0.10 | 0.7 | 70 | 70 | 0.809 | 0.810 | 0.049 | 22 | 22 | 0.816 | 0.806 | 0.053 |
|  |  |  | 0.9 | 54 | 54 | 0.806 | 0.807 | 0.044 | 18 | 18 | 0.835 | 0.830 | 0.053 |
|  | 0.5 | 0.01 | 0.7 | 82 | 80 | 0.805 | 0.792 | 0.051 | 26 | 26 | 0.824 | 0.779 | 0.062 |
|  |  |  | 0.9 | 64 | 64 | 0.807 | 0.802 | 0.050 | 20 | 20 | 0.812 | 0.776 | 0.052 |
|  |  | 0.10 | 0.7 | 110 | 106 | 0.806 | 0.807 | 0.051 | 34 | 34 | 0.820 | 0.804 | 0.056 |
|  |  |  | 0.9 | 86 | 84 | 0.803 | 0.806 | 0.053 | 28 | 26 | 0.804 | 0.793 | 0.053 |
| 100 | 0.1 | 0.01 | 0.7 | 36 | 36 | 0.805 | 0.802 | 0.059 | 12 | 12 | 0.834 | 0.817 | 0.053 |
|  |  |  | 0.9 | 28 | 28 | 0.803 | 0.783 | 0.044 | 10 | 10 | 0.857 | 0.842 | 0.053 |
|  |  | 0.10 | 0.7 | 36 | 36 | 0.820 | 0.809 | 0.051 | 12 | 12 | 0.848 | 0.827 | 0.049 |
|  |  |  | 0.9 | 28 | 28 | 0.820 | 0.806 | 0.048 | 10 | 10 | 0.871 | 0.866 | 0.048 |
|  | 0.5 | 0.01 | 0.7 | 46 | 44 | 0.806 | 0.788 | 0.058 | 14 | 14 | 0.818 | 0.762 | 0.054 |
|  |  |  | 0.9 | 36 | 36 | 0.814 | 0.778 | 0.064 | 12 | 12 | 0.842 | 0.777 | 0.051 |
|  |  | 0.10 | 0.7 | 58 | 56 | 0.801 | 0.785 | 0.052 | 18 | 18 | 0.816 | 0.776 | 0.055 |
|  |  |  | 0.9 | 46 | 46 | 0.815 | 0.812 | 0.055 | 14 | 14 | 0.809 | 0.770 | 0.050 |

**Appendix Table 6.** Estimated required number of clusters for HTE test by the direct inflation ($n_{0}$), the proposed formula under MCAR ($n_{1}$), the empirical type I error rate of the Wald test for HTE ($\psi$), the predicted power ($\phi_{pre}$) and empirical power ($\phi_{emp}$) of the HTE test with a binary individual-level effect modifier under MCAR. The effect size for power is $\delta\in\{0.25, 0.45\}$. The missingness ICC is $\tau=0.6$.

|  | | | | $\delta=0.25$ | | | | | $\delta=0.45$ | | | | |
| --- | --- | --- | --- | --- | --- | --- | --- | --- | --- | --- | --- | --- | --- |
| $m$ | $\rho_{x}$ | $\rho_{y\vert x}$ | $\pi$ | $n_{0}$ | $n_{1}$ | $\phi_{pre}$ | $\phi_{emp}$ | $\psi$ | $n_{0}$ | $n_{1}$ | $\phi_{pre}$ | $\phi_{emp}$ | $\psi$ |
| 20 | 0.1 | 0.01 | 0.7 | 174 | 174 | 0.802 | 0.800 | 0.055 | 54 | 54 | 0.804 | 0.785 | 0.055 |
|  |  |  | 0.9 | 136 | 136 | 0.804 | 0.803 | 0.051 | 42 | 42 | 0.804 | 0.806 | 0.052 |
|  |  | 0.10 | 0.7 | 172 | 172 | 0.803 | 0.812 | 0.048 | 54 | 54 | 0.809 | 0.800 | 0.057 |
|  |  |  | 0.9 | 134 | 134 | 0.803 | 0.802 | 0.050 | 42 | 42 | 0.809 | 0.814 | 0.054 |
|  | 0.5 | 0.01 | 0.7 | 186 | 184 | 0.801 | 0.788 | 0.055 | 58 | 58 | 0.809 | 0.801 | 0.052 |
|  |  |  | 0.9 | 146 | 144 | 0.801 | 0.795 | 0.045 | 46 | 46 | 0.814 | 0.788 | 0.042 |
|  |  | 0.10 | 0.7 | 242 | 232 | 0.800 | 0.777 | 0.047 | 76 | 72 | 0.802 | 0.779 | 0.052 |
|  |  |  | 0.9 | 188 | 186 | 0.802 | 0.789 | 0.052 | 58 | 58 | 0.806 | 0.808 | 0.054 |
| 50 | 0.1 | 0.01 | 0.7 | 72 | 72 | 0.810 | 0.810 | 0.047 | 22 | 22 | 0.806 | 0.794 | 0.054 |
|  |  |  | 0.9 | 56 | 56 | 0.809 | 0.806 | 0.048 | 18 | 18 | 0.824 | 0.804 | 0.051 |
|  |  | 0.10 | 0.7 | 70 | 70 | 0.809 | 0.788 | 0.052 | 22 | 22 | 0.816 | 0.803 | 0.050 |
|  |  |  | 0.9 | 54 | 54 | 0.806 | 0.805 | 0.053 | 18 | 18 | 0.835 | 0.820 | 0.058 |
|  | 0.5 | 0.01 | 0.7 | 82 | 80 | 0.801 | 0.791 | 0.053 | 26 | 26 | 0.821 | 0.772 | 0.058 |
|  |  |  | 0.9 | 64 | 64 | 0.806 | 0.801 | 0.053 | 20 | 20 | 0.811 | 0.761 | 0.060 |
|  |  | 0.10 | 0.7 | 110 | 106 | 0.805 | 0.795 | 0.048 | 34 | 34 | 0.819 | 0.794 | 0.052 |
|  |  |  | 0.9 | 86 | 84 | 0.803 | 0.798 | 0.043 | 28 | 26 | 0.804 | 0.773 | 0.058 |
| 100 | 0.1 | 0.01 | 0.7 | 36 | 36 | 0.804 | 0.791 | 0.054 | 12 | 12 | 0.833 | 0.806 | 0.050 |
|  |  |  | 0.9 | 28 | 28 | 0.803 | 0.785 | 0.047 | 10 | 10 | 0.857 | 0.843 | 0.064 |
|  |  | 0.10 | 0.7 | 36 | 36 | 0.820 | 0.812 | 0.053 | 12 | 12 | 0.848 | 0.823 | 0.050 |
|  |  |  | 0.9 | 28 | 28 | 0.820 | 0.802 | 0.046 | 10 | 10 | 0.871 | 0.858 | 0.055 |
|  | 0.5 | 0.01 | 0.7 | 46 | 44 | 0.801 | 0.788 | 0.051 | 14 | 14 | 0.813 | 0.717 | 0.061 |
|  |  |  | 0.9 | 36 | 36 | 0.813 | 0.793 | 0.058 | 12 | 12 | 0.841 | 0.780 | 0.063 |
|  |  | 0.10 | 0.7 | 58 | 56 | 0.800 | 0.797 | 0.049 | 18 | 18 | 0.816 | 0.755 | 0.054 |
|  |  |  | 0.9 | 46 | 46 | 0.815 | 0.803 | 0.051 | 14 | 14 | 0.809 | 0.768 | 0.048 |

**Appendix Table 7.** Estimated required number of clusters for HTE test by the direct inflation ($n_{0}$), the proposed formula under MCAR ($n_{1}$), the empirical type I error rate of the Wald test for HTE ($\psi$), the predicted power ($\phi_{pre}$) and empirical power ($\phi_{emp}$) of the HTE test with a binary individual-level effect modifier under MCAR. The effect size for power is $\delta\in\{0.25, 0.45\}$. The missingness ICC is $\tau=1$.

|  | | | | $\delta=0.25$ | | | | | $\delta=0.45$ | | | | |
| --- | --- | --- | --- | --- | --- | --- | --- | --- | --- | --- | --- | --- | --- |
| $m$ | $\rho_{x}$ | $\rho_{y\vert x}$ | $\pi$ | $n_{0}$ | $n_{1}$ | $\phi_{pre}$ | $\phi_{emp}$ | $\psi$ | $n_{0}$ | $n_{1}$ | $\phi_{pre}$ | $\phi_{emp}$ | $\psi$ |
| 20 | 0.1 | 0.01 | 0.7 | 174 | 174 | 0.802 | 0.789 | 0.050 | 54 | 54 | 0.804 | 0.795 | 0.049 |
|  |  |  | 0.9 | 136 | 136 | 0.803 | 0.799 | 0.048 | 42 | 42 | 0.804 | 0.781 | 0.050 |
|  |  | 0.10 | 0.7 | 172 | 172 | 0.803 | 0.793 | 0.048 | 54 | 54 | 0.809 | 0.792 | 0.054 |
|  |  |  | 0.9 | 134 | 134 | 0.803 | 0.809 | 0.049 | 42 | 42 | 0.809 | 0.807 | 0.039 |
|  | 0.5 | 0.01 | 0.7 | 186 | 186 | 0.802 | 0.799 | 0.047 | 58 | 58 | 0.806 | 0.780 | 0.051 |
|  |  |  | 0.9 | 146 | 146 | 0.805 | 0.807 | 0.059 | 46 | 46 | 0.813 | 0.785 | 0.054 |
|  |  | 0.10 | 0.7 | 242 | 236 | 0.803 | 0.793 | 0.051 | 76 | 74 | 0.809 | 0.794 | 0.053 |
|  |  |  | 0.9 | 188 | 186 | 0.801 | 0.787 | 0.050 | 58 | 58 | 0.805 | 0.801 | 0.049 |
| 50 | 0.1 | 0.01 | 0.7 | 72 | 72 | 0.809 | 0.798 | 0.050 | 22 | 22 | 0.805 | 0.761 | 0.048 |
|  |  |  | 0.9 | 56 | 56 | 0.809 | 0.805 | 0.053 | 18 | 18 | 0.824 | 0.824 | 0.045 |
|  |  | 0.10 | 0.7 | 70 | 70 | 0.809 | 0.812 | 0.043 | 22 | 22 | 0.816 | 0.789 | 0.053 |
|  |  |  | 0.9 | 54 | 54 | 0.806 | 0.809 | 0.048 | 18 | 18 | 0.835 | 0.821 | 0.060 |
|  | 0.5 | 0.01 | 0.7 | 82 | 82 | 0.805 | 0.782 | 0.050 | 26 | 26 | 0.815 | 0.760 | 0.056 |
|  |  |  | 0.9 | 64 | 64 | 0.805 | 0.791 | 0.048 | 20 | 20 | 0.809 | 0.760 | 0.062 |
|  |  | 0.10 | 0.7 | 110 | 106 | 0.803 | 0.767 | 0.057 | 34 | 34 | 0.818 | 0.776 | 0.050 |
|  |  |  | 0.9 | 86 | 84 | 0.802 | 0.784 | 0.048 | 28 | 26 | 0.803 | 0.781 | 0.060 |
| 100 | 0.1 | 0.01 | 0.7 | 36 | 36 | 0.803 | 0.785 | 0.048 | 12 | 12 | 0.832 | 0.777 | 0.054 |
|  |  |  | 0.9 | 28 | 28 | 0.803 | 0.804 | 0.047 | 10 | 10 | 0.856 | 0.834 | 0.046 |
|  |  | 0.10 | 0.7 | 36 | 36 | 0.820 | 0.815 | 0.055 | 12 | 12 | 0.848 | 0.807 | 0.049 |
|  |  |  | 0.9 | 28 | 28 | 0.820 | 0.814 | 0.046 | 10 | 10 | 0.871 | 0.846 | 0.049 |
|  | 0.5 | 0.01 | 0.7 | 46 | 46 | 0.813 | 0.790 | 0.056 | 14 | 14 | 0.807 | 0.705 | 0.054 |
|  |  |  | 0.9 | 36 | 36 | 0.811 | 0.784 | 0.062 | 12 | 12 | 0.840 | 0.767 | 0.062 |
|  |  | 0.10 | 0.7 | 58 | 58 | 0.813 | 0.789 | 0.054 | 18 | 18 | 0.815 | 0.741 | 0.054 |
|  |  |  | 0.9 | 46 | 46 | 0.815 | 0.795 | 0.052 | 14 | 14 | 0.809 | 0.750 | 0.051 |

**Appendix Table 8.** Estimated required number of clusters for HTE test by the direct inflation ($n_{0}$), the proposed formula under MCAR ($n_{1}$), and the proposed procedure under MAR ($n_{2}$), the empirical type I error rate of the Wald test for HTE ($\psi$), and predicted power ($\phi_{pre}^{,MAR}$) and empirical power ($\phi_{emp}^{MAR}$) of the HTE test with a continuous individual-level effect modifier under MAR. The effect size for power is of $\delta=\{0.1, 0.25\}$. The missingness ICC $\tau=0.3$.

|  | | | | $\delta=0.10$ | | | | | | $\delta=0.25$ | | | | | |
| --- | --- | --- | --- | --- | --- | --- | --- | --- | --- | --- | --- | --- | --- | --- | --- |
| $m$ | $\rho_{x}$ | $\rho_{y\vert x}$ | $\pi$ | $n_{0}$ | $n_{1}$ | $n_{2}$ | $\phi_{pre}^{MAR}$ | $\phi_{emp}^{MAR}$ | $\psi$ | $n_{0}$ | $n_{1}$ | $n_{2}$ | $\phi_{pre}^{MAR}$ | $\phi_{emp}^{MAR}$ | $\psi$ |
| 20 | 0.1 | 0.01 | 0.7 | 228 | 228 | 234 | 0.802 | 0.810 | 0.050 | 38 | 38 | 38 | 0.806 | 0.798 | 0.056 |
|  |  |  | 0.9 | 178 | 178 | 180 | 0.801 | 0.801 | 0.057 | 30 | 30 | 30 | 0.816 | 0.804 | 0.048 |
|  |  | 0.10 | 0.7 | 226 | 226 | 232 | 0.802 | 0.799 | 0.048 | 36 | 36 | 38 | 0.811 | 0.792 | 0.047 |
|  |  |  | 0.9 | 176 | 176 | 178 | 0.802 | 0.805 | 0.050 | 28 | 28 | 30 | 0.821 | 0.821 | 0.058 |
|  | 0.5 | 0.01 | 0.7 | 244 | 240 | 246 | 0.801 | 0.804 | 0.052 | 40 | 40 | 40 | 0.807 | 0.773 | 0.057 |
|  |  |  | 0.9 | 190 | 190 | 192 | 0.801 | 0.807 | 0.052 | 32 | 32 | 32 | 0.816 | 0.792 | 0.048 |
|  |  | 0.10 | 0.7 | 318 | 306 | 310 | 0.801 | 0.797 | 0.048 | 52 | 50 | 50 | 0.803 | 0.787 | 0.044 |
|  |  |  | 0.9 | 248 | 244 | 246 | 0.801 | 0.803 | 0.046 | 40 | 40 | 40 | 0.807 | 0.800 | 0.057 |
| 50 | 0.1 | 0.01 | 0.7 | 94 | 94 | 96 | 0.805 | 0.805 | 0.045 | 16 | 16 | 16 | 0.822 | 0.813 | 0.048 |
|  |  |  | 0.9 | 72 | 72 | 74 | 0.805 | 0.812 | 0.058 | 12 | 12 | 12 | 0.809 | 0.792 | 0.053 |
|  |  | 0.10 | 0.7 | 90 | 90 | 94 | 0.806 | 0.801 | 0.052 | 16 | 16 | 16 | 0.829 | 0.808 | 0.058 |
|  |  |  | 0.9 | 70 | 70 | 72 | 0.805 | 0.799 | 0.044 | 12 | 12 | 12 | 0.821 | 0.809 | 0.059 |
|  | 0.5 | 0.01 | 0.7 | 108 | 106 | 108 | 0.806 | 0.799 | 0.059 | 18 | 18 | 18 | 0.826 | 0.795 | 0.048 |
|  |  |  | 0.9 | 84 | 84 | 84 | 0.803 | 0.798 | 0.045 | 14 | 14 | 14 | 0.819 | 0.792 | 0.048 |
|  |  | 0.10 | 0.7 | 144 | 140 | 140 | 0.800 | 0.793 | 0.049 | 24 | 24 | 24 | 0.827 | 0.814 | 0.048 |
|  |  |  | 0.9 | 112 | 110 | 112 | 0.805 | 0.799 | 0.047 | 18 | 18 | 18 | 0.807 | 0.798 | 0.058 |
| 100 | 0.1 | 0.01 | 0.7 | 48 | 48 | 50 | 0.817 | 0.809 | 0.056 | 8 | 8 | 8 | 0.811 | 0.794 | 0.053 |
|  |  |  | 0.9 | 38 | 38 | 38 | 0.810 | 0.802 | 0.053 | 6 | 6 | 6 | 0.804 | 0.796 | 0.050 |
|  |  | 0.10 | 0.7 | 46 | 46 | 48 | 0.813 | 0.823 | 0.048 | 8 | 8 | 8 | 0.826 | 0.813 | 0.053 |
|  |  |  | 0.9 | 36 | 36 | 36 | 0.806 | 0.803 | 0.051 | 6 | 6 | 6 | 0.821 | 0.824 | 0.049 |
|  | 0.5 | 0.01 | 0.7 | 60 | 58 | 60 | 0.811 | 0.785 | 0.061 | 10 | 10 | 10 | 0.826 | 0.783 | 0.058 |
|  |  |  | 0.9 | 48 | 46 | 46 | 0.800 | 0.801 | 0.053 | 8 | 8 | 8 | 0.829 | 0.776 | 0.059 |
|  |  | 0.10 | 0.7 | 76 | 76 | 76 | 0.806 | 0.781 | 0.049 | 14 | 12 | 12 | 0.800 | 0.791 | 0.049 |
|  |  |  | 0.9 | 60 | 60 | 60 | 0.809 | 0.791 | 0.053 | 10 | 10 | 10 | 0.824 | 0.814 | 0.048 |

**Appendix Table 9.** Estimated required number of clusters for HTE test by the direct inflation ($n_{0}$), the proposed formula under MCAR ($n_{1}$), and the proposed procedure under MAR ($n_{2}$), the empirical type I error rate of the Wald test for HTE ($\psi$), and predicted power ($\phi_{pre}^{,MAR}$) and empirical power ($\phi_{emp}^{MAR}$) of the HTE test with a continuous individual-level effect modifier under MAR. The effect size for power is of $\delta=\{0.1, 0.25\}$. The missingness ICC $\tau=0.6$.

|  | | | | $\delta=0.10$ | | | | | | $\delta=0.25$ | | | | | |
| --- | --- | --- | --- | --- | --- | --- | --- | --- | --- | --- | --- | --- | --- | --- | --- |
| $m$ | $\rho_{x}$ | $\rho_{y\vert x}$ | $\pi$ | $n_{0}$ | $n_{1}$ | $n_{2}$ | $\phi_{pre}^{MAR}$ | $\phi_{emp}^{MAR}$ | $\psi$ | $n_{0}$ | $n_{1}$ | $n_{2}$ | $\phi_{pre}^{MAR}$ | $\phi_{emp}^{MAR}$ | $\psi$ |
| 20 | 0.1 | 0.01 | 0.7 | 228 | 228 | 232 | 0.802 | 0.804 | 0.048 | 38 | 38 | 38 | 0.810 | 0.790 | 0.047 |
|  |  |  | 0.9 | 178 | 178 | 180 | 0.803 | 0.802 | 0.045 | 30 | 30 | 30 | 0.818 | 0.801 | 0.052 |
|  |  | 0.10 | 0.7 | 226 | 226 | 230 | 0.802 | 0.806 | 0.053 | 36 | 36 | 38 | 0.815 | 0.805 | 0.059 |
|  |  |  | 0.9 | 176 | 176 | 176 | 0.800 | 0.802 | 0.058 | 28 | 28 | 30 | 0.823 | 0.808 | 0.051 |
|  | 0.5 | 0.01 | 0.7 | 244 | 242 | 244 | 0.800 | 0.797 | 0.046 | 40 | 40 | 40 | 0.810 | 0.775 | 0.053 |
|  |  |  | 0.9 | 190 | 190 | 190 | 0.800 | 0.794 | 0.048 | 32 | 32 | 32 | 0.817 | 0.780 | 0.049 |
|  |  | 0.10 | 0.7 | 318 | 312 | 310 | 0.800 | 0.800 | 0.043 | 52 | 50 | 50 | 0.804 | 0.794 | 0.049 |
|  |  |  | 0.9 | 248 | 246 | 246 | 0.802 | 0.806 | 0.054 | 40 | 40 | 40 | 0.808 | 0.800 | 0.055 |
| 50 | 0.1 | 0.01 | 0.7 | 94 | 94 | 94 | 0.802 | 0.798 | 0.045 | 16 | 16 | 16 | 0.821 | 0.794 | 0.050 |
|  |  |  | 0.9 | 72 | 72 | 74 | 0.806 | 0.791 | 0.049 | 12 | 12 | 12 | 0.811 | 0.792 | 0.053 |
|  |  | 0.10 | 0.7 | 90 | 90 | 92 | 0.801 | 0.788 | 0.053 | 16 | 16 | 16 | 0.833 | 0.820 | 0.054 |
|  |  |  | 0.9 | 70 | 70 | 72 | 0.808 | 0.813 | 0.058 | 12 | 12 | 12 | 0.823 | 0.809 | 0.054 |
|  | 0.5 | 0.01 | 0.7 | 108 | 106 | 106 | 0.801 | 0.795 | 0.048 | 18 | 18 | 18 | 0.825 | 0.770 | 0.058 |
|  |  |  | 0.9 | 84 | 84 | 84 | 0.805 | 0.797 | 0.052 | 14 | 14 | 14 | 0.820 | 0.786 | 0.054 |
|  |  | 0.10 | 0.7 | 144 | 142 | 140 | 0.802 | 0.795 | 0.051 | 24 | 24 | 24 | 0.827 | 0.815 | 0.053 |
|  |  |  | 0.9 | 112 | 112 | 112 | 0.806 | 0.798 | 0.055 | 18 | 18 | 18 | 0.808 | 0.808 | 0.050 |
| 100 | 0.1 | 0.01 | 0.7 | 48 | 48 | 48 | 0.802 | 0.799 | 0.051 | 8 | 8 | 8 | 0.815 | 0.781 | 0.057 |
|  |  |  | 0.9 | 38 | 38 | 38 | 0.812 | 0.808 | 0.053 | 6 | 6 | 6 | 0.808 | 0.795 | 0.061 |
|  |  | 0.10 | 0.7 | 46 | 46 | 46 | 0.802 | 0.800 | 0.052 | 8 | 8 | 8 | 0.827 | 0.800 | 0.053 |
|  |  |  | 0.9 | 36 | 36 | 36 | 0.808 | 0.812 | 0.052 | 6 | 6 | 6 | 0.823 | 0.804 | 0.057 |
|  | 0.5 | 0.01 | 0.7 | 60 | 60 | 60 | 0.810 | 0.813 | 0.053 | 10 | 10 | 10 | 0.825 | 0.767 | 0.058 |
|  |  |  | 0.9 | 48 | 46 | 46 | 0.800 | 0.784 | 0.051 | 8 | 8 | 8 | 0.829 | 0.769 | 0.058 |
|  |  | 0.10 | 0.7 | 76 | 76 | 76 | 0.807 | 0.813 | 0.054 | 14 | 12 | 12 | 0.803 | 0.788 | 0.050 |
|  |  |  | 0.9 | 60 | 60 | 60 | 0.810 | 0.808 | 0.051 | 10 | 10 | 10 | 0.825 | 0.815 | 0.060 |

**Appendix Table 10.** Estimated required number of clusters for HTE test by the direct inflation ($n_{0}$), the proposed formula under MCAR ($n_{1}$), and the proposed procedure under MAR ($n_{2}$), the empirical type I error rate of the Wald test for HTE ($\psi$), and predicted power ($\phi_{pre}^{,MAR}$) and empirical power ($\phi_{emp}^{MAR}$) of the HTE test with a binary individual-level effect modifier under MAR. The effect size for power is of $\delta=\{0.25, 0.45\}$. The missingness ICC $\tau=0.05$.

|  | | | | $\delta=0.25$ | | | | | | $\delta=0.45$ | | | | | |
| --- | --- | --- | --- | --- | --- | --- | --- | --- | --- | --- | --- | --- | --- | --- | --- |
| $m$ | $\rho_{x}$ | $\rho_{y\vert x}$ | $\pi$ | $n_{0}$ | $n_{1}$ | $n_{2}$ | $\phi_{pre}^{MAR}$ | $\phi_{emp}^{MAR}$ | $\psi$ | $n_{0}$ | $n_{1}$ | $n_{2}$ | $\phi_{pre}^{MAR}$ | $\phi_{emp}^{MAR}$ | $\psi$ |
| 20 | 0.1 | 0.01 | 0.7 | 174 | 174 | 166 | 0.804 | 0.803 | 0.049 | 54 | 54 | 52 | 0.810 | 0.795 | 0.052 |
|  |  |  | 0.9 | 136 | 136 | 134 | 0.805 | 0.792 | 0.055 | 42 | 42 | 42 | 0.811 | 0.819 | 0.046 |
|  |  | 0.10 | 0.7 | 172 | 172 | 164 | 0.803 | 0.802 | 0.051 | 54 | 54 | 52 | 0.812 | 0.804 | 0.053 |
|  |  |  | 0.9 | 134 | 134 | 132 | 0.803 | 0.793 | 0.050 | 42 | 42 | 42 | 0.816 | 0.825 | 0.047 |
|  | 0.5 | 0.01 | 0.7 | 186 | 182 | 174 | 0.803 | 0.787 | 0.051 | 58 | 58 | 54 | 0.805 | 0.772 | 0.056 |
|  |  |  | 0.9 | 146 | 144 | 142 | 0.803 | 0.796 | 0.044 | 46 | 46 | 44 | 0.807 | 0.791 | 0.049 |
|  |  | 0.10 | 0.7 | 242 | 230 | 220 | 0.801 | 0.798 | 0.046 | 76 | 72 | 68 | 0.801 | 0.803 | 0.051 |
|  |  |  | 0.9 | 188 | 186 | 184 | 0.803 | 0.799 | 0.052 | 58 | 58 | 58 | 0.811 | 0.804 | 0.060 |
| 50 | 0.1 | 0.01 | 0.7 | 72 | 70 | 68 | 0.809 | 0.814 | 0.048 | 22 | 22 | 22 | 0.826 | 0.819 | 0.054 |
|  |  |  | 0.9 | 56 | 56 | 54 | 0.802 | 0.804 | 0.047 | 18 | 18 | 18 | 0.833 | 0.821 | 0.048 |
|  |  | 0.10 | 0.7 | 70 | 70 | 66 | 0.804 | 0.804 | 0.047 | 22 | 22 | 22 | 0.834 | 0.834 | 0.053 |
|  |  |  | 0.9 | 54 | 54 | 54 | 0.813 | 0.819 | 0.045 | 18 | 18 | 18 | 0.842 | 0.834 | 0.052 |
|  | 0.5 | 0.01 | 0.7 | 82 | 80 | 76 | 0.806 | 0.803 | 0.045 | 26 | 26 | 24 | 0.815 | 0.788 | 0.056 |
|  |  |  | 0.9 | 64 | 64 | 62 | 0.803 | 0.801 | 0.047 | 20 | 20 | 20 | 0.817 | 0.784 | 0.057 |
|  |  | 0.10 | 0.7 | 110 | 106 | 102 | 0.807 | 0.793 | 0.053 | 34 | 34 | 32 | 0.813 | 0.796 | 0.053 |
|  |  |  | 0.9 | 86 | 84 | 84 | 0.808 | 0.810 | 0.060 | 28 | 26 | 26 | 0.809 | 0.778 | 0.063 |
| 100 | 0.1 | 0.01 | 0.7 | 36 | 36 | 34 | 0.802 | 0.805 | 0.048 | 12 | 12 | 12 | 0.851 | 0.843 | 0.056 |
|  |  |  | 0.9 | 28 | 28 | 28 | 0.808 | 0.807 | 0.049 | 10 | 10 | 10 | 0.863 | 0.858 | 0.054 |
|  |  | 0.10 | 0.7 | 36 | 36 | 34 | 0.815 | 0.819 | 0.043 | 12 | 12 | 12 | 0.863 | 0.860 | 0.057 |
|  |  |  | 0.9 | 28 | 28 | 28 | 0.825 | 0.820 | 0.049 | 10 | 10 | 10 | 0.877 | 0.874 | 0.054 |
|  | 0.5 | 0.01 | 0.7 | 46 | 44 | 42 | 0.810 | 0.801 | 0.053 | 14 | 14 | 14 | 0.838 | 0.793 | 0.052 |
|  |  |  | 0.9 | 36 | 36 | 36 | 0.820 | 0.793 | 0.049 | 12 | 12 | 12 | 0.850 | 0.800 | 0.058 |
|  |  | 0.10 | 0.7 | 58 | 56 | 56 | 0.815 | 0.801 | 0.057 | 18 | 18 | 18 | 0.828 | 0.789 | 0.054 |
|  |  |  | 0.9 | 46 | 46 | 44 | 0.802 | 0.774 | 0.049 | 14 | 14 | 14 | 0.816 | 0.787 | 0.054 |

**Appendix Table 11.** Estimated required number of clusters for HTE test by the direct inflation ($n_{0}$), the proposed formula under MCAR ($n_{1}$), and the proposed procedure under MAR ($n_{2}$), the empirical type I error rate of the Wald test for HTE ($\psi$), and predicted power ($\phi_{pre}^{,MAR}$) and empirical power ($\phi_{emp}^{MAR}$) of the HTE test with a binary individual-level effect modifier under MAR. The effect size for power is of $\delta=\{0.25, 0.45\}$. The missingness ICC $\tau=0.3$.

|  | | | | $\delta=0.25$ | | | | | | $\delta=0.45$ | | | | | |
| --- | --- | --- | --- | --- | --- | --- | --- | --- | --- | --- | --- | --- | --- | --- | --- |
| $m$ | $\rho_{x}$ | $\rho_{y\vert x}$ | $\pi$ | $n_{0}$ | $n_{1}$ | $n_{2}$ | $\phi_{pre}^{MAR}$ | $\phi_{emp}^{MAR}$ | $\psi$ | $n_{0}$ | $n_{1}$ | $n_{2}$ | $\phi_{pre}^{MAR}$ | $\phi_{emp}^{MAR}$ | $\psi$ |
| 20 | 0.1 | 0.01 | 0.7 | 174 | 174 | 166 | 0.801 | 0.787 | 0.050 | 54 | 54 | 52 | 0.807 | 0.800 | 0.057 |
|  |  |  | 0.9 | 136 | 136 | 134 | 0.804 | 0.806 | 0.052 | 42 | 42 | 42 | 0.811 | 0.811 | 0.048 |
|  |  | 0.10 | 0.7 | 172 | 172 | 166 | 0.805 | 0.799 | 0.047 | 54 | 54 | 52 | 0.810 | 0.807 | 0.060 |
|  |  |  | 0.9 | 134 | 134 | 132 | 0.803 | 0.792 | 0.045 | 42 | 42 | 42 | 0.816 | 0.810 | 0.051 |
|  | 0.5 | 0.01 | 0.7 | 186 | 184 | 176 | 0.801 | 0.798 | 0.050 | 58 | 58 | 56 | 0.815 | 0.799 | 0.044 |
|  |  |  | 0.9 | 146 | 144 | 142 | 0.802 | 0.780 | 0.043 | 46 | 46 | 44 | 0.806 | 0.774 | 0.049 |
|  |  | 0.10 | 0.7 | 242 | 234 | 224 | 0.802 | 0.792 | 0.042 | 76 | 72 | 70 | 0.807 | 0.800 | 0.052 |
|  |  |  | 0.9 | 188 | 186 | 184 | 0.802 | 0.789 | 0.051 | 58 | 58 | 58 | 0.810 | 0.790 | 0.058 |
| 50 | 0.1 | 0.01 | 0.7 | 72 | 72 | 68 | 0.805 | 0.810 | 0.051 | 22 | 22 | 22 | 0.822 | 0.808 | 0.059 |
|  |  |  | 0.9 | 56 | 56 | 54 | 0.802 | 0.805 | 0.055 | 18 | 18 | 18 | 0.832 | 0.823 | 0.052 |
|  |  | 0.10 | 0.7 | 70 | 70 | 66 | 0.801 | 0.805 | 0.050 | 22 | 22 | 22 | 0.831 | 0.824 | 0.053 |
|  |  |  | 0.9 | 54 | 54 | 54 | 0.812 | 0.805 | 0.049 | 18 | 18 | 18 | 0.842 | 0.833 | 0.055 |
|  | 0.5 | 0.01 | 0.7 | 82 | 80 | 76 | 0.801 | 0.796 | 0.052 | 26 | 26 | 24 | 0.811 | 0.782 | 0.059 |
|  |  |  | 0.9 | 64 | 64 | 62 | 0.802 | 0.801 | 0.049 | 20 | 20 | 20 | 0.816 | 0.773 | 0.052 |
|  |  | 0.10 | 0.7 | 110 | 106 | 102 | 0.803 | 0.796 | 0.047 | 34 | 34 | 32 | 0.810 | 0.796 | 0.045 |
|  |  |  | 0.9 | 86 | 84 | 84 | 0.807 | 0.791 | 0.050 | 28 | 26 | 26 | 0.808 | 0.787 | 0.050 |
| 100 | 0.1 | 0.01 | 0.7 | 36 | 36 | 36 | 0.819 | 0.819 | 0.053 | 12 | 12 | 12 | 0.846 | 0.825 | 0.052 |
|  |  |  | 0.9 | 28 | 28 | 28 | 0.807 | 0.813 | 0.048 | 10 | 10 | 10 | 0.862 | 0.843 | 0.053 |
|  |  | 0.10 | 0.7 | 36 | 36 | 34 | 0.813 | 0.822 | 0.054 | 12 | 12 | 12 | 0.860 | 0.848 | 0.052 |
|  |  |  | 0.9 | 28 | 28 | 28 | 0.824 | 0.828 | 0.046 | 10 | 10 | 10 | 0.876 | 0.858 | 0.058 |
|  | 0.5 | 0.01 | 0.7 | 46 | 44 | 42 | 0.805 | 0.797 | 0.046 | 14 | 14 | 14 | 0.835 | 0.804 | 0.064 |
|  |  |  | 0.9 | 36 | 36 | 36 | 0.819 | 0.806 | 0.050 | 12 | 12 | 12 | 0.849 | 0.789 | 0.064 |
|  |  | 0.10 | 0.7 | 58 | 58 | 56 | 0.812 | 0.809 | 0.050 | 18 | 18 | 18 | 0.825 | 0.802 | 0.055 |
|  |  |  | 0.9 | 46 | 46 | 44 | 0.801 | 0.781 | 0.045 | 14 | 14 | 14 | 0.816 | 0.774 | 0.049 |

**Appendix Table 12.** Estimated required number of clusters for HTE test by the direct inflation ($n_{0}$), the proposed formula under MCAR ($n_{1}$), and the proposed procedure under MAR ($n_{2}$), the empirical type I error rate of the Wald test for HTE ($\psi$), and predicted power ($\phi_{pre}^{,MAR}$) and empirical power ($\phi_{emp}^{MAR}$) of the HTE test with a binary individual-level effect modifier under MAR. The effect size for power is of $\delta=\{0.25, 0.45\}$. The missingness ICC $\tau=0.6$.

|  | | | | $\delta=0.25$ | | | | | | $\delta=0.45$ | | | | | |
| --- | --- | --- | --- | --- | --- | --- | --- | --- | --- | --- | --- | --- | --- | --- | --- |
| $m$ | $\rho_{x}$ | $\rho_{y\vert x}$ | $\pi$ | $n_{0}$ | $n_{1}$ | $n_{2}$ | $\phi_{pre}^{MAR}$ | $\phi_{emp}^{MAR}$ | $\psi$ | $n_{0}$ | $n_{1}$ | $n_{2}$ | $\phi_{pre}^{MAR}$ | $\phi_{emp}^{MAR}$ | $\psi$ |
| 20 | 0.1 | 0.01 | 0.7 | 174 | 174 | 168 | 0.801 | 0.801 | 0.053 | 54 | 54 | 52 | 0.802 | 0.797 | 0.051 |
|  |  |  | 0.9 | 136 | 136 | 134 | 0.803 | 0.797 | 0.055 | 42 | 42 | 42 | 0.809 | 0.801 | 0.044 |
|  |  | 0.10 | 0.7 | 172 | 172 | 168 | 0.804 | 0.810 | 0.046 | 54 | 54 | 52 | 0.806 | 0.813 | 0.047 |
|  |  |  | 0.9 | 134 | 134 | 132 | 0.801 | 0.793 | 0.057 | 42 | 42 | 42 | 0.814 | 0.812 | 0.044 |
|  | 0.5 | 0.01 | 0.7 | 186 | 184 | 180 | 0.804 | 0.791 | 0.060 | 58 | 58 | 56 | 0.808 | 0.799 | 0.048 |
|  |  |  | 0.9 | 146 | 144 | 142 | 0.800 | 0.790 | 0.054 | 46 | 46 | 44 | 0.803 | 0.773 | 0.051 |
|  |  | 0.10 | 0.7 | 242 | 238 | 228 | 0.804 | 0.804 | 0.051 | 76 | 74 | 72 | 0.810 | 0.806 | 0.046 |
|  |  |  | 0.9 | 188 | 188 | 184 | 0.800 | 0.782 | 0.057 | 58 | 58 | 58 | 0.809 | 0.809 | 0.057 |
| 50 | 0.1 | 0.01 | 0.7 | 72 | 72 | 70 | 0.813 | 0.792 | 0.046 | 22 | 22 | 22 | 0.817 | 0.807 | 0.052 |
|  |  |  | 0.9 | 56 | 56 | 54 | 0.800 | 0.808 | 0.052 | 18 | 18 | 18 | 0.831 | 0.819 | 0.054 |
|  |  | 0.10 | 0.7 | 70 | 70 | 68 | 0.810 | 0.810 | 0.056 | 22 | 22 | 22 | 0.825 | 0.820 | 0.046 |
|  |  |  | 0.9 | 54 | 54 | 54 | 0.810 | 0.798 | 0.053 | 18 | 18 | 18 | 0.841 | 0.842 | 0.055 |
|  | 0.5 | 0.01 | 0.7 | 82 | 82 | 78 | 0.805 | 0.790 | 0.054 | 26 | 26 | 24 | 0.805 | 0.759 | 0.052 |
|  |  |  | 0.9 | 64 | 64 | 64 | 0.810 | 0.791 | 0.052 | 20 | 20 | 20 | 0.814 | 0.770 | 0.057 |
|  |  | 0.10 | 0.7 | 110 | 108 | 104 | 0.804 | 0.803 | 0.051 | 34 | 34 | 32 | 0.806 | 0.781 | 0.057 |
|  |  |  | 0.9 | 86 | 84 | 84 | 0.806 | 0.809 | 0.048 | 28 | 26 | 26 | 0.806 | 0.776 | 0.055 |
| 100 | 0.1 | 0.01 | 0.7 | 36 | 36 | 36 | 0.816 | 0.818 | 0.051 | 12 | 12 | 12 | 0.843 | 0.824 | 0.057 |
|  |  |  | 0.9 | 28 | 28 | 28 | 0.806 | 0.802 | 0.052 | 10 | 10 | 10 | 0.861 | 0.851 | 0.056 |
|  |  | 0.10 | 0.7 | 36 | 36 | 34 | 0.809 | 0.801 | 0.042 | 12 | 12 | 12 | 0.856 | 0.843 | 0.052 |
|  |  |  | 0.9 | 28 | 28 | 28 | 0.823 | 0.810 | 0.049 | 10 | 10 | 10 | 0.875 | 0.851 | 0.054 |
|  | 0.5 | 0.01 | 0.7 | 46 | 46 | 44 | 0.812 | 0.802 | 0.050 | 14 | 14 | 14 | 0.827 | 0.760 | 0.058 |
|  |  |  | 0.9 | 36 | 36 | 36 | 0.818 | 0.806 | 0.051 | 12 | 12 | 12 | 0.847 | 0.789 | 0.064 |
|  |  | 0.10 | 0.7 | 58 | 58 | 56 | 0.808 | 0.785 | 0.048 | 18 | 18 | 18 | 0.821 | 0.790 | 0.051 |
|  |  |  | 0.9 | 46 | 46 | 44 | 0.800 | 0.795 | 0.051 | 14 | 14 | 14 | 0.814 | 0.768 | 0.049 |
